# The process of culturally adapting the Healthy Beginnings early obesity prevention program for Arabic and Chinese mothers in Australia

**DOI:** 10.1186/s12889-021-10270-5

**Published:** 2021-02-04

**Authors:** Sarah Marshall, Sarah Taki, Penny Love, Yvonne Laird, Marianne Kearney, Nancy Tam, Louise A. Baur, Chris Rissel, Li Ming Wen

**Affiliations:** 1 Sydney School of Public Health, University of Sydney, Camperdown, NSW 2006 Australia; 2 Health Promotion Unit, Population Health Research and Evaluation Hub, Sydney Local Health District, Level 9, King George V Building, Missenden Road, Camperdown, NSW 2050 Australia; 3 The National Health and Medical Research Council Centre of Research Excellence in the Early Prevention of Obesity in Childhood (EPOCH CRE), Sydney, Australia; 4 Institute for Physical Activity and Nutrition, School of Exercise and Nutrition Sciences, Deakin University, Waurn Ponds, Victoria 3216 Australia; 5 Sydney Medical School, University of Sydney, Camperdown, NSW 2006 Australia

**Keywords:** Prevention, Childhood obesity, Health promotion, Nutrition, Infant, Culture, Ethnicity

## Abstract

**Background:**

Behavioural interventions for the early prevention of childhood obesity mostly focus on English-speaking populations in high-income countries. Cultural adaptation is an emerging strategy for implementing evidence-based interventions among different populations and regions. This paper describes the initial process of culturally adapting Healthy Beginnings, an evidence-based early childhood obesity prevention program, for Arabic and Chinese speaking migrant mothers and infants in Sydney, Australia.

**Methods:**

The cultural adaptation process followed the Stages of Cultural Adaptation theoretical model and is reported using the Framework for Reporting Adaptations and Modifications-Enhanced. We first established the adaptation rationale, then considered program underpinnings and the core components for effectiveness. To inform adaptations, we reviewed the scientific literature and engaged stakeholders. Consultations included focus groups with 24 Arabic and 22 Chinese speaking migrant mothers and interviews with 20 health professionals. With input from project partners, bi-cultural staff and community organisations, findings informed cultural adaptations to the content and delivery features of the Healthy Beginnings program.

**Results:**

Program structure and delivery mode were retained to preserve fidelity (i.e. staged nurse calls with key program messages addressing modifiable obesity-related behaviours: infant feeding, active play, sedentary behaviours and sleep). Qualitative analysis of focus group and interview data resulted in descriptive themes concerning cultural practices and beliefs related to infant obesity-related behaviours and perceptions of child weight among Arabic and Chinese speaking mothers. Based on the literature and local study findings, cultural adaptations were made to recruitment approaches, staffing (bi-cultural nurses and project staff) and program content (modified call scripts and culturally adapted written health promotion materials).

**Conclusions:**

This cultural adaptation of Healthy Beginnings followed an established process model and resulted in a program with enhanced relevance and accessibility among Arabic and Chinese speaking migrant mothers. This work will inform the future cultural adaptation stages: testing, refining, and trialling the culturally adapted Healthy Beginnings program to assess acceptability, feasibility and effectiveness.

**Supplementary Information:**

The online version contains supplementary material available at 10.1186/s12889-021-10270-5.

## Background

Nutrition and physical activity in the first 2000 days of a child’s life - from conception until 5 years - are important predictors of healthy weight during childhood and later life [1, 2]. This critical period for childhood obesity prevention is a global priority for establishing lifelong health [3]. Childhood obesity rates are increasing globally, with multiple early risk factors identified, including maternal characteristics and modifiable behaviours [4].

Diverse ethnic and cultural minority populations experience higher childhood obesity prevalence rates and show differences in early life behavioural risk factors [5, 6]. Cultural beliefs, practices and acculturation after migration can contribute to differences in childhood obesity prevalence [7]. Structural and environmental factors also play a role [8]. Cultural feeding practices and traditional foods can be protective of obesity development [9].

In recent years, research interventions to support healthy early life behaviours have increased, with many targeting individual-level behaviours of parents and infants in high-income countries [10–12]. However, cultural minority populations are largely underrepresented. Meta-analysis of four randomised controlled trials (RCTs) for early life obesity prevention in Australia and New Zealand [13–16] found a significant reduction of child body mass index (BMI) z-score and improvements in selected behaviours at age 18–24 months [17]. These RCTs were developed primarily for English-speaking populations.

With global migration increasing, predominantly toward high-income countries [18], there is a need to ensure programs supporting early healthy growth and development are culturally relevant and accessible to families from culturally and linguistically diverse backgrounds.

### Cultural adaptation

Cultural adaptation of an intervention refers to modifications made to become more suitable to a new target population considering culture, language and context [19]. Balance is sought between adhering to the intervention (fidelity) and modifying the intervention for cultural relevance (fit) [20]. Meta-analyses and reviews of culturally adapted childhood obesity prevention interventions show acceptability and improved outcomes among target populations [21–23].

Culturally adapted health promotion interventions promoting healthy eating and physical activity largely focus on adults, with few targeting children aged under 5 years [24]. International examples include the ‘Fit 5 Kids’ television reduction program for Latino children aged 3–5 years from America [25] and the HAPPY (Healthy and Active Parenting Programme for Early Years) program for South-Asian origin pregnant women and infants from the United Kingdom [26]. These interventions developed for English-speaking populations were adapted for cultural groups and showed positive outcomes and acceptance.

Over the past decade, several reviews have advanced theoretical approaches for cultural adaptation [27–30], including a recent review of cultural adaptation guidance [31]. Cultural adaptation can be guided by process and content models, offering instruction on ‘how’ and ‘what’ to culturally adapt. Process models present steps for adaptation (for an example, see Kumpfer [32]), whereas content frameworks guide which program components to adapt (see Bernal’s Ecological Validity Model [19]). Reviews of culturally adapted interventions call for more rigorous and consistent use of theory and reporting standards [21, 23, 30].

### The Healthy Beginnings program and the current study

Healthy Beginnings is an effective, nurse-delivered early obesity prevention intervention offering individual support to women during pregnancy and their child’s first years of life [13]. The program focusses on known obesity-related early life behaviours: infant nutrition, active play including ‘tummy time’ (supervised time awake in the prone position) and sedentary behaviours [33, 34] at set time-points aligned with child development milestones.

The first Healthy Beginnings RCT offered nurse home-visits to mothers in socioeconomically disadvantaged areas of Sydney [13] and effectively reduced prevalence of overweight and obesity and improved feeding practices among children in the intervention group at age 2 years [35]. To increase reach and cost-effectiveness, a subsequent RCT - Communicating Healthy Beginnings Advice by Telephone (CHAT) - offered the same nurse support via phone calls or SMS (short message service) with written booklets to reinforce content [36] and also showed effectiveness for achieving target behaviours [37]. In both trials, eligibility criteria included the ability to communicate in English.

In Sydney, Australia, there is increasing migratory population growth; over one third (35.8%) of the population speaks a language other than English at home [38], most commonly Mandarin and Arabic [39]. Australian children from Middle Eastern and Asian backgrounds experience disproportionate prevalence rates of overweight and obesity [6]. To focus early obesity prevention efforts, we explored the cultural adaptation of Healthy Beginnings.

This paper reports the process of culturally adapting Healthy Beginnings delivered by telephone among Arabic and Chinese speaking migrant mothers in Sydney, Australia and summarises the adapted intervention.

## Methods

### Design and context

The Healthy Beginnings cultural adaptation project commenced in January 2018, led by the Health Promotion Unit, Sydney Local Health District. The project was supported by an existing grant and the mainstream Healthy Beginnings resources and governance structures. The state and local policy environments were also favourable, with an emphasis on reducing inequalities, preventing childhood obesity and promoting health in the first 2000 days of life [40–43].

The project team comprised senior researchers and highly qualified staff, including Arabic and Chinese bi-cultural workers and research leadership. This team led the cultural adaptations with input from bi-cultural staff from local health districts and cultural community organisations. Additional file 1 describes the project team and lists the contributors.

We used the Framework for Reporting Adaptations and Modifications-Enhanced (FRAME) by Stirman and colleagues [44, 45] to guide the reporting of this cultural adaptation. FRAME guides the reporting of program modifications but also contextual factors, such as the setting and who was involved. This is important for replicability and unpacking outcomes. FRAME components are discussed throughout, and the populated tools are in Additional file 2.

The Ethics Review Committee of Sydney Local Health District granted ethics approval for this project (reference X18–0049, HREC/18/RPAH/82).

### Framework for the cultural adaptation process

We based our process on Barrera and colleagues’ Stages of Cultural Adaptation stepwise model [46] which includes: 1) information gathering; 2) preliminary adaptations; 3) testing; 4) refining; and 5) conducting a trial. Barrera’s model was chosen as it was developed from consolidating literature to culturally adapt behavioural health interventions. While this model includes reference to establishing rationale and assessing the existing program, it lacks instruction on initial explorations [31]. When culturally adapting an intervention, there is a need to review the underpinning theories and worldviews so as not to assume universality [47], as well as determine the core components deemed essential to achieving the desired program outcomes [48, 49]. To clarify our rationale and critical assessment of the mainstream Healthy Beginnings program, we included this as our first step.

**Fig. 1:**
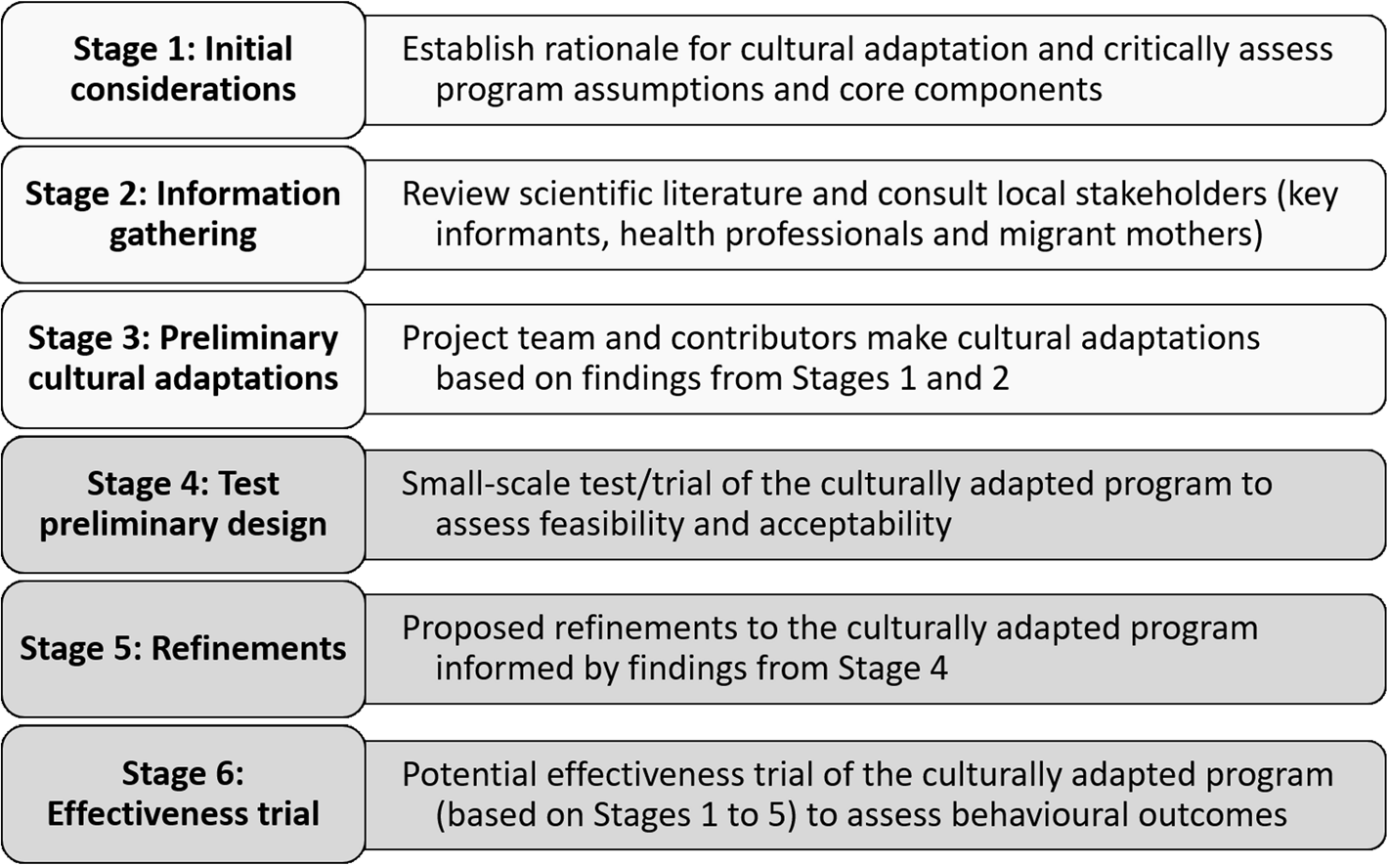
Overview of the process for culturally adapting the Healthy Beginnings program

This paper reports the adaptation stages of 1) initial considerations; 2) information gathering and 3) preliminary cultural adaptations, outlined in Fig. 1. Our subsequent stages, 4) testing and 5) refining the adapted intervention, will be reported elsewhere. The final stage, 6) trial of the intervention to assess effectiveness, forms the proposed direction for this work. Additional file 3 presents the overall project and timeline.

#### Stage 1: initial considerations

We proposed cultural adaptations to Healthy Beginnings to improve relevance, reach and engagement with migrant mothers primarily speaking Arabic or Chinese and living in Sydney. Our justifications for cultural adaptations were guided by a) ineffective engagement and non-significant outcomes for Arabic and Chinese speaking cultural groups; b) programmatic mismatches; and c) unique factors related to obesity-related early life behaviours [20, 28, 46]. Culture is multi-dimensional: including ethnicity, gender, socioeconomic position, identity and contextual experiences [50]. We considered this when defining the target groups.

We examined the Healthy Beginnings program theory of change, logic model, and behaviour change techniques to consider underpinning worldviews and determine the core components likely to account for effectiveness. Behaviour change techniques are replicable elements of behavioural interventions that result in behaviour change, characterised by behaviour change technique taxonomy [51].

#### Stage 2: information gathering

The aim of Stage 2 was to collect current and local information from literature and stakeholders to determine the preliminary adaptations to Healthy Beginnings.

##### Review of the literature

A review of published literature was conducted during February to July 2018 to identify a) early obesity prevention interventions among mainstream and minority groups to assess core program components and; b) subcultural group differences in modifiable risk factors (infant feeding, active play and sleep) specific to Arabic and Chinese speaking migrants, particularly in Australia.

##### Qualitative consultations with local staff and community members

During project set-up, the project team spoke to key informants, seeking information to guide the preliminary adaptations. Informants included key staff from the local health districts working with Arabic and Chinese speaking migrant families, the Healthy Beginnings management committee and partner community organisations.

The formal consultations included focus groups with Arabic and Chinese speaking mothers and individual interviews with child and family health professionals. These consultations primarily aimed to understand practices, attitudes and beliefs about Healthy Beginnings’ key content areas - infant feeding, active play, sedentary behaviours and sleep - and to invite feedback on draft adapted written material. The details of data collection are described in full elsewhere [52], in a separate but complementary study aimed to understand support for migrant mothers’ infant feeding; results highlight the importance of family, bi-cultural doctors and culturally sensitive maternal and child health care. We present the methods here in brief.

Arabic and Chinese speaking migrant mothers of young children and health professionals working with Arabic and/or Chinese speaking migrant families were recruited in Sydney from July until November 2018. All mothers and staff who responded to promotional material were provided with the study information in English, Arabic or Simplified Chinese. All participants could ask questions with the research team at any time (in English, Arabic or Chinese). All participants completed a consent form and brief demographic survey at the time of the focus group or interview.

Focus groups and interviews followed pilot-tested and refined semi-structured guides developed for this study. See Additional file 4 for the brief demographic surveys and guides used. Questions focussed on access to health services, infant feeding, physical activity and attitudes toward infant weight. The focus groups with mothers included an opportunity to comment on the draft adapted Healthy Beginnings booklets. The Chinese language focus groups were facilitated by a bi-cultural project team member (NT). The Arabic language focus groups were facilitated by three female bi-cultural health staff. Focus group audio files were professionally transcribed verbatim in Arabic and Simplified Chinese, checked for accuracy by bilingual staff, then professionally translated to English and checked again. All staff interviews were facilitated in English by a project staff member and doctoral candidate (SM), with audio files then professionally transcribed and checked for accuracy.

In this study, we undertook a descriptive thematic analysis using the framework method [53] from a critical realist methodological stance (where there is an assumed reality in the data expressed by participants). NVivo 12 software (QSR International; 2019) was used to manage the data. In summary, the analysis included familiarisation, data-derived semantic coding, development of a data matrix, and generation of themes. The intention of this analysis was not to explore underlying latent themes, but instead to describe explicit content aligned with program key content to then practically apply this in Stage 2. Derived themes were grouped under domains: key behaviours - infant feeding, active play, sedentary behaviours, sleep; and perceptions related to infant weight. The primary author (SM) led the analysis, with mentoring from qualitative experts (ST and PL) and with ongoing input from co-authors.

#### Stage 3: preliminary cultural adaptations

After initial considerations and based on the information gathered, aspects of program content and delivery features were assessed for adaptations intended to improve cultural appropriateness and acceptability among Arabic and Chinese speaking migrant mothers.

Program delivery features that were deemed core to achieving program outcomes were not adapted. Other delivery features were considered for potential adaptations and focussed on addressing cultural mismatches from the mainstream program and issues identified during the first stages.

Content changes included tailoring messages, nurse call scripts and written resources. Changes were made at both the surface and deep structural level, considering not only surface-level translations and images, but also deeper-level changes to reflect values and beliefs [54]. The framework by Kreuter et al. [55] further specifies surface and deep cultural components and was used to describe the content changes made to Healthy Beginnings.

To culturally adapt the Healthy Beginnings written resources (booklets and SMS), we followed four steps based on guidelines for adaptation of health resources [56, 57]. In brief, the steps included 1) preliminary adaptation in English based on information gathered, including adapted graphics and readability assessment (using Flesch-Kincaid Grade Level); 2) translation by staff accredited by the Australian National Accreditation Authority for Translators and Interpreters then quality checking for accuracy and preservation of linguistic meanings by professional linguists and bi-cultural team members; 3) pilot testing of the adapted and translated draft versions with community members and bi-cultural team members; 4) integration of results and final proofing by bi-cultural team members. The booklets were professionally designed during pilot testing and finalisation.

### Financial costs of the cultural adaptation process

Financial costs of the adaptation process are rarely reported. During the cultural adaptation process presented here, the financial costs of activities were recorded. Results are presented as cost of Australian dollars according to main items and total amount.

## Results

### Stage 1: initial considerations

#### Rationale for culturally adapting Healthy Beginnings

In the mainstream Healthy Beginnings, mothers with limited English language proficiency were excluded, therefore were not effectively engaged and program efficacy could not be assessed. Program mismatches included participant characteristics (e.g. language, migration status, cultural factors) and delivery staff (detailed in Additional file 5). In addition, analysis of Healthy Beginnings data at child age 2 years highlighted maternal country of origin (born outside Australia) as a significant predictor of poorer child diet quality [58] and child BMI [59].

In this cultural adaptation, sub-cultural grouping was based on Arabic and Chinese language as a component of ethnicity as well as common cross-cultural experiences of migration and acculturation. Studies find that migrant mothers in high-income English-speaking countries commonly experience being ‘between cultures’ when accessing maternity care [60, 61] and making infant feeding decisions [62].

#### Program theories and worldviews

The mainstream Healthy Beginnings program is grounded in the socio-ecological model of health, where broader societal factors are recognised to influence individual behaviour and health. The program was developed based on established behaviour change theories: Social Learning Theory and the Health Belief Model [13]. Social Learning Theory is commonly and successfully used in early obesity prevention interventions [63]. There have been criticisms of the use of the Health Belief Model in cross-cultural research due to the individualistic nature and omission of social and cultural factors [64], therefore the Social Learning Theory was an important complement for this cultural adaptation. The project team considered the worldviews of the socio-ecological model and the two behaviour change theories to allow for Westernised, individualistic approaches but also collectivist and family-oriented approaches that align with aspects of Arabic and Chinese cultures.

#### Program core components

From examining the Healthy Beginnings program theories, logic model and behaviour change techniques, the core components were deemed to be 1) key content areas related to infant feeding, active play and sleep, 2) existing behaviour change techniques and 3) delivery mode via staged individual nurse calls and written materials sent by mail.

The primary aim of the Healthy Beginnings program is to establish healthy behaviours in early life to prevent obesity corresponding to key milestones of child development. Primary behavioural outcomes relate to increased breastfeeding rate and duration, appropriate timing of introducing complementary foods (solids), increased rate of practising ‘tummy time’, increased rate of using a cup and drinking water, and increased intake of fruits/vegetables. Therefore, we retained the key content areas from the mainstream program that relate to known early life obesity risk factors [36] and align with Australian and international infant feeding and activity guidelines.

Thirteen behaviour change techniques were employed in the original Healthy Beginnings RCT [65]. The subsequent phone-based Healthy Beginnings (CHAT) included behaviour change techniques: goal setting (behaviour), problem solving, review of behaviour goals, feedback on behaviour, social support (unspecified), instruction on how to perform a behaviour, information about health consequences and credible source. Six of these behaviour change techniques (all except goal setting and credible source) have been associated with effective health-professional delivered interventions for childhood obesity prevention [11]. These were deemed core components and were retained.

The program key content areas and behaviour change techniques were delivered by individual nurse phone calls. Nurse calls were staged according to program content areas, infant age and developmental milestones (one during the third trimester of pregnancy, then at infant age 1, 3, 5 months), and followed a script/prompt tailored to the individual participants with corresponding written material. See Table 2 for a summary of the delivery features. Considering the nurse calls were the primary means of delivering the content and associated with strongest behavioural outcomes in the mainstream program [37], the project team deemed the mode of delivery to also be core to the program.

### Stage 2: information gathering

#### Findings from literature reviews

- Early obesity prevention interventions among mainstream and minority groups

Systematic reviews related to early obesity prevention behavioural interventions were important for establishing context and need for this cultural adaptation. At the time of literature searching, there were few published interventions for obesity prevention among parents and children aged 0–5 years that accounted for sub-cultural differences or targeted minority populations [10, 66, 67]. Those that did address disadvantage or diversity mostly adjusted staff and recruitment methods, but culturally tailored content was infrequent and not reported in detail.

Antenatal and postnatal support delivered via phone can be effective [68]. Concurrent modes of delivery are beneficial alongside individual counselling for breastfeeding interventions [69]. No reviews were identified that investigated mode of intervention delivery among minority groups. Broadly, parents from culturally and linguistically diverse backgrounds with infants born in Australia are less likely to utilise health services [70] and the practical and emotional supports they receive are different from mothers born in Australia [71], emphasising the relevance of individual phone support.

b) Infant feeding, active play and sleep among Arabic and Chinese speaking populations

Literature was identified related to modifiable obesity-related behaviours in early life among Arabic and Chinese speaking migrant populations on infant feeding, but not specific to infant active play or sleep.

A key review of Chinese migrant mothers’ infant feeding practices globally highlighted high rates of breastfeeding initiation, mixed-feeding with formula and earlier introduction of complementary foods [72]. Previous Australian research found that Arabic and Chinese speaking migrant mothers had longer breastfeeding durations [73, 74], yet lower exclusive breastfeeding at 12 weeks when compared to Australian-born mothers [74]. Recent analysis of the Australian National Infant Feeding Survey data found that infants of Chinese-born mothers were younger when they first consumed infant formula, water-based drinks and fruit juice but older when they first ate complementary foods compared to infants of Australian-born mothers [75]. Qualitative findings among Chinese migrant mothers in Australia described cultural beliefs and influences on infant feeding practices after migration, such as the role of grandparents and perceptions of insufficient breastmilk leading to introduction of infant formula [76]. Chinese cultural beliefs at the time of child birth were also described, for example ‘yin and yang’ balances [77].

There was less literature available regarding Arabic speaking migrants and infant feeding practices. One review found infant feeding practices in the Middle Eastern region (as opposed to *after* migration) included early mixed feeding with formula or other fluids and early introduction of complementary foods [78]. Possible explanatory cultural factors were not explored in depth. A study of experiences of Arabic migrant mothers in Canada highlighted high breastfeeding rates and strong family support [79]. No recent published research with Arabic speaking migrant mothers within Australia was available.

#### Findings from qualitative consultations with local community members and staff

Initial conversations between the project lead investigator, the mainstream Healthy Beginnings project staff and management committee were essential in determining a need for the Healthy Beginnings among Arabic and Chinese speaking migrant mothers. While the mainstream program engaged mothers with culturally and linguistically diverse backgrounds, the program was limited to the English language and some mothers found this difficult [80].

The formative research included six focus groups with a total of 24 Arabic and 22 Chinese speaking mothers and individual interviews with 20 child and family health professionals in Sydney. All participating mothers were born in Chinese and Arabic speaking countries, except two who were born in Australia. More Arabic speaking mothers were newly arrived (< 10 years in Australia) (*n*=14) compared to the Chinese speaking mothers (*n*=7). Health professionals were mostly child and family health nurses (*n*=15) with over 10 years of professional experience (*n*=14). Most of the health professionals only spoke English at home (*n*=14), but some also spoke Arabic, Mandarin or Cantonese languages (*n*=6).

Analysis of the focus group and interview data resulted in themes related to participant experiences and perceptions of obesity-related behaviours among Arabic and Chinese speaking migrant mothers in Australia. Table 1 provides a summary of the main findings and indicates the source of data for each theme. The themes are described using illustrative quotes. Further illustrative quotes are presented in Additional file 6.

**Table 1:** Qualitative analysis findings from consultations with Arabic and Chinese speaking migrant mothers and health professionals

Domains and sub-themes	Arabic speaking mothers	Chinese speaking mothers	Health professionals
**Domain 1: Beliefs and practices related to infant feeding, active play, sedentary behaviours, sleep**			
Confinement practices important for recovery after birth	x	x	x
Breastfeeding as part of the social norm and expected	x		x
Not enough breastmilk and formula use for reassurance		x	x
Feeding as helpful for weight gain, sleep and settling	x	x	x
Confusion with ideal timing of complementary feeding	x	x	x
‘Tummy time’ as a new concept	x	x	x
The floor is not seen as a place for new babies to play	x	x	x
‘Tummy time’ as a risk for new babies		x	x
Interactions/play with babies as natural	x	x	x
Dilemmas of screen use	x	x	x
Screens to distract baby while feeding			x
**Domain 2: Perceptions related to child weight**			
A bigger baby as a healthier baby	x	x	x
Infant overweight as not a concern			x

##### Beliefs and practices related to infant obesity-related behaviours

Confinement practices were described by many mothers in both language groups; a time of resting, approximately 40 days after birth, important for recovery and for care of the newborn. Mothers sometimes referred to this as an old tradition, but still practised this to varying degrees and often to fulfil family wishes. Mothers sometimes found that health services were not always culturally accepting of these beliefs. Health professionals perceived that grandmothers influenced this decision and practice. Only some health professionals indicated that they adjusted their service provision to show understanding and respect for this practice.

*“It depends on the mother themselves whether they follow the guideline here or that in their own country. You know, they will feel different here.”* - Multicultural community health worker, 6 years, Mandarin and English

Arabic speaking mothers talked about breastfeeding being supported and a norm among their cultural community. This perception was not expressed in the same way among the Chinese speaking mothers, where breastfeeding was important but not necessarily an expectation. Health professionals were also mindful of these predominant beliefs, and some recognised the influence of Islam and religion in the practice to breastfeed for longer.

*"Frankly, non-one advised me to bottle-feed, all people encouraged me to breastfeed." -* Arabic speaking mother, Focus group 4

Chinese speaking mothers and health professionals expressed perceptions of insufficient breastmilk production and resultant concerns of inadequate infant feeding and growth. This perception was not evident among the Arabic speaking mothers. For the Chinese speaking mothers, this concern often resulted in top-up feeding with infant formula or sometimes the introduction of complementary foods. Health professionals perceived a lack of confidence among mothers related to the felt inability to produce enough milk but also the thought that breastmilk alone may not be nutritionally complete.

*"At that time, my mother was helping me with the baby, so when the baby was hungry, you would not necessarily have enough breast milk."* - Chinese speaking mother, Focus group 1

There was a tendency to feed baby with less responsiveness to hunger and fullness cues and more in response to ‘filling up.’ This is linked to the view that a bigger baby is healthier, but also to help to ease crying, calm the baby and improve sleep. From the mothers’ perspective, this was often a pressure from grandparents rather than the mothers themselves.

*"If they whinge, you give them food or milk because they will stop complaining, even if they might be crying over something else."* - Child and family health nurse, 23 years, English

Arabic and Chinese speaking mothers spoke of introducing complementary foods at varying times and there was much mixed information about the best time to start. Mothers often mentioned that family, friends, sometimes health professionals suggested starting earlier. Some mothers were aware of the guideline to wait until around 6 months, others saw the earlier introduction of complementary foods as a positive sign of a developing baby.

*“You can then puree this thing for him to have a taste. If the child shows no interest, then you can maybe wait. If he shows interest at a fairly early stage, you can try it early with him.” -* Chinese speaking mother, Focus group 1

Arabic and Chinese speaking mothers had mixed knowledge and practices of ‘tummy time’ and physical activity  with babies, depending on their exposure to this concept. Some mothers said that this was not taught in their home countries, and they first heard the concept from health services in Australia. There were beliefs expressed in both Arabic and Chinese groups that the floor could be dirty and was not an ideal place for baby. Health professionals often endorsed the use of a play mat on the floor. Some Chinese speaking mothers expressed differing views from their grandparents who saw ‘tummy time’ as unsuitable for very young babies; there was concern that the position could injure the baby.

*“We don’t put the baby on the floor [in Syria]. …This is the first time I know about it”* - Arabic speaking mother, Focus group 5

For many of the Arabic and Chinese speaking mothers, interactions with babies were a natural part of motherhood, and not necessarily motivated by child development. Interactions included singing, massage and play using facial expressions. Mothers’ views and practices related to screen use were mixed. Many Arabic and Chinese speaking mothers felt that early screen use was not good for babies (harmful for eyes and brain) and some were aware that early screen use was not recommended. Some mothers used screens as a learning tool, for settling infants, occupying babies during house duties and used screens increasingly with multiple children. Some mothers felt this was inevitable given the technological environment, and they expressed a sense of guilt for doing this. While mothers did not talk about use of screens during feeding, some health professionals described non-responsive feeding while babies were distracted with screens; this was not always seen as related to cultural background, but a broader societal norm.

*“At the early stage, his eyes are still developing, it will have a bigger impact, so you should try to delay [using screens]. Then again, there are many apps that are really helpful to their study. However, the later you introduce this the better.”* - Chinese speaking mother, Focus group 2

##### Perceptions related to child weight

Sub-themes ‘bigger baby as a healthier baby’ and ‘infant overweight as not a concern’ were interrelated. Arabic and Chinese speaking mothers both referred to perceptions that a bigger baby is a healthy baby. Health professionals perceived this as a strong belief among both communities. While mothers referred to this, there was indication that this view was more strongly held by grandparents and not necessarily by the mothers themselves. Mothers spoke about other signs of health for their baby such as being happy, alert, playful and physically well. Health professionals spoke about this as a difficult belief to manage when giving advice as they perceived that parents did not see overweight as a concern.

*“The weight is important. It’s healthy. If they are skinny, then people will ask is there anything wrong with the baby.” -* Child and family health nurse, 16 years, Cantonese and English

### Stage 3: preliminary cultural adaptation

#### Culturally adapted Healthy Beginnings delivery features

Characteristics of delivery staff and recruitment approaches were critical adaptations for addressing cultural mismatches from the mainstream program. Table 2 presents delivery features of the mainstream Healthy Beginnings compared with the culturally adapted program.

**Table 2:** Delivery features of the mainstream and culturally adapted Healthy Beginnings program delivered by telephone

	Mainstream Healthy Beginnings – English speaking mothers	Culturally adapted Healthy Beginnings - Arabic and Chinese speaking migrant mothers
**Recruitment**		
**Promotional material**	Flyer in English developed by project staff.	Flyer adapted by bi-cultural project staff, with culturally relevant images and translated text focussing on infant growth and development.
**Strategies**	Recruitment via flyers at antenatal clinics and promotion from midwives.	Recruitment face-to-face with bi-cultural research staff and medical professional translator at antenatal clinics and groups.
**Delivery features**		
**Program components**		
Nurse phone calls	Participant-led discussion including goal setting, with script/prompts based on program key content, infant age and developmental milestones, individualised to each mother. Call script/prompts developed by health professionals for Australian mothers.	Participant-led discussion including goal setting, with script/prompts based on program key message, infant age and developmental milestones, individualised to each mother. Call script/prompts culturally adapted and translated by health professionals, bi-cultural workers, translators. Script/prompts adapted to cultural factors (identified in information gathering stage) and bi-cultural nurse experience.
Written Healthy Beginnings booklets	Healthy Beginnings information booklets aligned with key content areas and timing of nurse calls. Developed by health professionals for Australian mothers and families. Mailed to home address with option to email.	Healthy Beginnings information booklets aligned with key content areas and timing of nurse calls. Adapted and translated by health professionals, bi-cultural workers, professional translators and Arabic and Chinese community members. Adapted to cultural factors (identified in information gathering stage). Mailed to home address with option to email. Additional breastfeeding promotional poster sent.
Written complementary material	Relevant supporting resources available in English (e.g. physical activity and healthy eating guidelines for children).	Relevant supporting resources available in Arabic and Simplified Chinese (e.g. translations of Australian Guide to Healthy Eating, local Arabic or Chinese play groups).
Phone text messages	Aligned with staged calls with opportunity for mothers to reply. SMS sent twice per week for 4 weeks. Personalised by name, according to age and feeding mode. Developed by health professionals for Australian mothers and families.	Aligned with staged calls with opportunity for mothers to reply. SMS sent twice per week for 4 weeks. Personalised by name, according to age and feeding mode. Adapted and translated by health professionals, bi-cultural workers and translators. Key content retained but adapted to cultural factors (identified in information gathering stage).
**Delivery staff**	Female child and family health nurse, in English language.	Female bi-cultural child and family health nurse, in Arabic or Chinese languages.
**Program audience**	Individual mothers	Individual mothers
**Program location**	Phone calls to participant’s home	Phone calls to participant’s home
**Program duration**	24 months	6 months
**Session number**	8 staged intervention phone calls, with additional calls as required	4 staged intervention phone calls, with additional calls as required
**Session frequency**	One call during the third trimester of pregnancy, then at infant age 1, 3, 5, 9, 12, 18 and 24 months	Once call during the third trimester of pregnancy, then at infant age 1, 3, 5 months

Female bi-cultural child and family health nurses who spoke Arabic or Chinese languages were employed. The delivery of individualised calls from bi-lingual nurses was important for providing information related to individual circumstances and diversity within language groups. Recruitment strategies included recruitment flyers with culturally relevant images of babies of Arabic or Chinese ethnicity. The text was written in Arabic or Simplified Chinese and emphasised healthy growth and development as opposed to obesity prevention, as this was identified in the information gathering as more likely to resonate and motivate engagement with the program.

#### Culturally adapted Healthy Beginnings content

Main content adaptations were based on the literature findings and qualitative research. To describe the surface and deep structural level changes made to Healthy Beginnings content, five main categories - peripheral, evidential, linguistic, constituent-involving and sociocultural approaches [55] are described below. Table 3 outlines the main content for each staged phone call and corresponding written material for the mainstream and the culturally adapted Healthy Beginnings. Additional file 7 shows sample content from the booklets, which are available to download from the project website [81].

**Table 3:** Main content of the mainstream and culturally adapted Healthy Beginnings program

Timing and key content areas / Behaviour targets	Main content		
	Mainstream Healthy Beginnings program [36]	Culturally adapted Healthy Beginnings – Arabic speaking migrant mothers	Culturally adapted Healthy Beginnings – Chinese speaking migrant mothers
Antenatal (third trimester) Sustaining breastfeeding / best-practice formula feeding	▪ Breastfeeding guidelines ▪ Health benefits of breastfeeding and strategies to overcome barriers associated with breastfeeding	▪ Breastfeeding guidelines. Reinforce with support of community ▪ Benefits of breastfeeding and colostrum; Breastmilk production in first weeks. ▪ Family and social support ▪ Information about accessing free health services and interpreters	▪ Breastfeeding guidelines. ▪ Benefits of breastfeeding and colostrum; Breastmilk production in first weeks; address concerns of not enough breastmilk for baby. ▪ Family and social support ▪ Information about accessing free health services and interpreters
0–2 months Sustaining breastfeeding / best-practice formula Timing of solid food introduction Promote active play ‘Tummy time’ Response to child cues: hunger, satiety	▪ Rapid response to women with problems initiating breastfeeding after childbirth, especially women who delivered by caesarean section ▪ Advice on establishment of breastfeeding pattern ▪ Management of feeding problems ▪ Infant feeding cues ▪ Baby feed, play, sleep cycle ▪ ‘tummy time’ for babies	▪ Rapid response to women with problems initiating breastfeeding ▪ Advice on establishment of breastfeeding pattern ▪ Management of problems ▪ Reinforce no other fluids or foods needed until around 6 months (e.g. formula and water) ▪ Infant feeding cues ▪ ‘tummy time’ for babies; with increased information about what, why and how ▪ Baby feed, play, sleep cycle; infant crying is normal. ▪ Family and social support; sharing care with fathers and family, and emotional support ▪ Information about accessing free health services and interpreters	▪ Rapid response to women with problems initiating breastfeeding ▪ Advice on establishment of breastfeeding pattern ▪ Management of problems – addressing any concerns of milk quality and/or quantity. ▪ Reinforce no other fluids or foods needed until around 6 months (e.g. formula and water) ▪ Infant feeding cues ▪ ‘tummy time’ for babies; with increased information about what, why and how. ▪ Baby feed, play, sleep cycle; infant crying is normal. ▪ Family and social support; sharing care with fathers and family and emotional support ▪ Information about accessing free health services and interpreters
2–4 months Sustaining breastfeeding / best-practice formula Timing of solid food introduction Promote active play ‘Tummy time’ Response to child cues: hunger, satiety	▪ Advice on establishment of breastfeeding patterns ▪ Management of problems ▪ ‘tummy time’ for babies ▪ Introduction of solids at around 6 months ▪ Encourage mothers going back to work to continue breastfeeding	▪ Advice on establishment of breastfeeding patterns ▪ Management of problems; describe signs that baby is getting enough breastmilk ▪ ‘tummy time’ for babies ▪ Introduction of solids at around 6 months ▪ Baby feed, play, sleep cycle; Sleep and settling techniques ▪ Family and social support ▪ Parenting; looking after mother and father’s emotional health ▪ Information about accessing free health services and interpreters; introduction to family child health nurse	▪ Advice on establishment of breastfeeding patterns ▪ Management of problems; describe signs that baby is getting enough breastmilk ▪ ‘tummy time’ for babies ▪ Introduction of solids at around 6 months, emphasise includes water/fluids too ▪ Encourage mothers going back to work to continue breastfeeding and offer strategies. ▪ Baby feed, play, sleep cycle; Sleep and settling techniques ▪ Family and social support ▪ Parenting; looking after mother and father’s emotional health ▪ Information about accessing free health services and interpreters; introduction to family child health nurse
4–6 months Solid food introduction Healthy food choices Promote active play ‘Tummy time’ and no screen use	▪ Reinforce breastfeeding pattern, Management of problems ▪ ‘tummy time’ for babies ▪ Introduction of solids from 6 months ▪ Encourage mothers going back to work to continue breastfeeding	▪ Reinforce breastfeeding pattern ▪ Management of problems ▪ ‘tummy time’ for babies ▪ Introduction of solids from 6 months; visually illustrating age-appropriate food textures ▪ Learning to eat and making a mess ▪ Following hunger and fullness cues ▪ Encourage mothers going back to work to continue breastfeeding and offer strategies ▪ Family and social support ▪ Parent-child interactions; importance of play for mental & emotional development ▪ Information about accessing free health services and interpreters	▪ Reinforce breastfeeding pattern, Management of problems ▪ ‘tummy time’ for babies ▪ Introduction of solids from 6 months; visually illustrating age-appropriate food textures ▪ Learning to eat and making a mess ▪ Following hunger and fullness cues ▪ Encourage mothers going back to work to continue breastfeeding and offer strategies ▪ Family and social support ▪ Parent-child interactions; importance of play for mental & emotional development ▪ Information about accessing free health services and interpreters

##### Peripheral approaches

For the written Healthy Beginnings booklets, peripheral features including colours, images and photographs were culturally adapted to suit Arabic and Chinese cultures. The professional graphic designers assisted in maintaining the Healthy Beginnings brand and incorporating adaptations to the aesthetic and the images.

Photos from the mainstream booklets were changed for ethnically representative photos. In the Arabic booklets, photos were selected to depict women both with and without a hijab to be religiously indistinct. Food images were culturally relevant, for example lentils, rice and flat bread. The Chinese booklets included food images such as rice, congee and wok-fried vegetables. The project team took care to select stock photos that appeared to be real-life (rather than staged) and represented a range of home situations. In addition, more visuals were used to explain concepts (opposed to text in the mainstream booklets), for example images explaining ‘tummy time’ and breastmilk needs in the first week of life.

##### Linguistic strategies

The participant intervention materials (booklets and SMS text) were written in Arabic and Simplified Chinese. The adaptation and translation process required extensive involvement from bi-cultural project staff, external professional staff and community members. The preliminary adaptation in English was sixth to eighth grade using the Flesch-Kincaid Grade Level, which equates to easy to average readability. Preservation of linguistic meanings was essential. For example, in both languages there is no direct translation for ‘tummy time’ or ‘solids’ (complementary foods). These words along with others were changed to plain English in Step 1 of the written material adaptation then carefully translated.

##### Evidential approaches

This approach relates to data, testimonials and narratives specific to Arabic and Chinese speaking migrant mothers to raise awareness of the health issues. Given the information gathering findings that childhood obesity was not perceived as a problem, other health aspects were emphasised. For example, the health benefits of breastfeeding were elaborated (e.g. building a close bond with the mother and reducing the risk of infection) and the risk of tooth decay from fruit juice or other sweet drinks was added. Additionally, Chinese speaking families placed importance on the information being the latest global scientific evidence and this was highlighted in the booklets, for example, stating “World Health Organisation recommends giving only breastmilk until baby is 6 months old”.

##### Constituent-involving strategies

Female bi-cultural child and family health nurses, identifying as and able to speak Arabic or Chinese languages, were employed in place of English-speaking child and family health nurses. The aim was to leverage the ‘insider’ knowledge of the culture and community. The shared understanding of the broader environment for Arabic and Chinese speaking migrant mothers and their experiences of infant feeding, active play, sedentary behaviours and sleep were anticipated to increase engagement, relevance and acceptability with mothers during the individualised nurse calls.

##### Sociocultural approaches

This approach relates to discussing the health issue in the context of specific social and/or cultural characteristics of the group. Arabic and Chinese cultural values and beliefs were incorporated, such as using ‘hot and cold’ foods from traditional Chinese medicine and presenting indoor physical activities acknowledging traditional confinement practices for recovery after birth among both cultures. Arabic cultural characteristics presented the project team and bi-cultural staff with greater challenges due to the religious and ethnic diversity among Arabic speaking countries. A connecting characteristic is the centrality and importance of the family unit, which is consistent among Arabic and Chinese cultures. While the program primarily aimed to influence mothers’ and infants’ behaviours, the role of family was promoted throughout the content. For example, information and images about sharing infant care with fathers and grandparents. Acknowledging the importance of mental health, content related to mothers’ emotional well-being was also enhanced.

### Financial costs of the cultural adaptation process

The financial costs for activities approximated $67,300 Australian dollars in direct spending. Conducting the focus groups (refreshments, venue hire, childcare, translation of participant research documents, facilitation) approximated $7500. Professional transcription and translation of focus groups and interviews added research costs ($21,400). Costs for the written materials (SMS messages and booklets) included the professional translations and typesetting ($8400) and professional design of the booklets ($30,000). Throughout the project, staff time and input from contributors were essential, however this in-kind support could not be quantified retrospectively.

## Discussion

This study reported the process undertaken for culturally adapting the Healthy Beginnings program for Arabic and Chinese speaking migrant mothers in Sydney, Australia. Essential initial steps included establishing the rationale, a critical review of the program's theoretical underpinnings and core components, review of the published literature and consultations with local target communities and health professionals. This informed program modifications for improved cultural relevance.

To our knowledge, this is the first cultural adaptation of an evidence-based intervention for early obesity prevention among migrant families with infants in Australia. There are international examples of early childhood obesity prevention interventions specially developed for minority populations, but this is a markedly different process from cultural adaptation of an existing intervention. Cultural adaptation offers promise for leveraging an existing evidence-based intervention for use among different populations. A strength of this study is the use of established cultural adaptation models to guide the stepwise process and content changes and use of a framework for thorough reporting.

Our findings build understanding of obesity-related behaviours among infants of Arabic and Chinese speaking migrant mothers. The themes developed relating to infant feeding practices and beliefs are aligned with qualitative findings among both Chinese [76] and Arabic speaking migrant mothers in Australia [82]; particularly the perceptions of not enough breastmilk and formula use for reassurance among Chinese speaking mothers, and breastfeeding as part of the social norm among Arabic speaking mothers. Recent findings among Chinese migrant mothers in Australia have highlighted the role of motherhood identity in mothers’ breastfeeding experiences [83]. In our study, thematic analysis findings related to infant active play, screen use and sleep among Arabic and Chinese speaking migrant mothers are novel, with no other similar published findings. Understanding, at a semantic level, that the concept of ‘tummy time’ was new to many and that the floor was not necessarily seen as a place for babies, were important contextual findings for informing the content adaptations to Healthy Beginnings.

### Cultural adaptation learning and challenges

Ongoing stakeholder involvement is recommended during the cultural adaptation process [27, 28, 31]. We strived to achieve this, seeking integration of ‘top-down’ (using evidence from mainstream Healthy Beginnings) and ‘bottom-up’ (input from stakeholders) approaches to inform the cultural adaptations [46]. While we attempted to form a steering committee including representative stakeholders, we faced challenges including scheduling, logistics and pre-determined project milestones tied to the funding period. The recognised challenge of ensuring a consensus process for cultural adaptation decisions [46] was also a consideration. Instead, final decisions were made by the project team which included, and was led by, bi-cultural team members, with input from Arabic and Chinese speaking migrant mothers and contributors including cultural community organisations. There is no agreement on ideal stakeholder engagement [31] and future adaptation projects should ensure sufficient time and resources prior to the funding period to establish relationships and form mechanisms for collaboration.

In this study, the cultural adaptations were based on language groups, Arabic and Chinese-Mandarin. Using language as a grouping can be criticised as there are many subcultural groupings within ethnic and language groups, particularly across countries and religions [28, 46]. The additional experience of migration to Australia is a common factor that narrowed our two language groupings. The challenge lies in achieving a population health promotion intervention for migrant populations with broad enough reach to increase impact and inclusivity, while not assuming or portraying homogeneity. In adapting the intervention, the project team made efforts to address this challenge - through use of inclusive linguistic translations and images, not generalising advice, and involving mothers who migrated to Australia from Arabic and Chinese speaking countries.

While the theoretical program underpinnings were critically deliberated, deeper cultural values and belief systems (e.g. consideration of religions, collectivism, cultural identity) could have been further embedded. The challenge of striking a balance between fidelity to the mainstream intervention core components and fit with target communities remains a debate [20, 47]. Our approach to cultural adaptations was conservative, aligning with the mainstream Healthy Beginnings’ underpinning theories. Modifying the program conceptual framework to incorporate worldviews and cultural factors among Arabic and Chinese speaking migrant communities could strengthen this adaptation and the subsequent stages of testing, refining, and trialling the intervention.

The time and resources required for culturally adapting a behavioural intervention are significant. This study leveraged in-kind support from the mainstream Healthy Beginnings program resources and partnerships. Even with this strong foundation, the time and resources required were extensive; both time and resources being acknowledged as essential for stakeholder engagement in research [84]. It is possible to reduce some of these costs, for example professional design of the intervention materials. Cost should not be part of the rationale to exclude culturally and linguistically diverse participants - nor is this equitable - but it is a necessary consideration when culturally adapting an intervention.

### Future directions

Subsequent steps of the Stages of Cultural Adaptation process model [46] include testing and refining the culturally adapted Healthy Beginnings program, then conducting an effectiveness trial. The testing and refining stages will address acceptability and feasibility of the culturally adapted intervention. A trial stage will determine what (if any) cultural adaptations are necessary to achieve the infant behavioural and weight outcomes - a question that often remains unanswered in cultural adaptation studies. Research designs accounting for multiple versions of the adapted program offer a promising strategy to unpack this question [30]. In the context of multicultural countries with high migration, cultural adaptation efforts could investigate pragmatic and adaptive ways of incorporating heterogeneity to maximise reach among minority cultural groups [28, 50].

### Limitations

Formative research with migrant mothers and health professionals focussed on Healthy Beginnings key content areas due to the limited literature regarding infant obesity-related behaviours among Arabic and Chinese speaking migrant families in Australia and locally in Sydney. Migrant mothers were invited to comment on draft program written materials, however, were not asked about the program structure or delivery. Instead, project contributors provided input on the appropriateness of the program for Arabic and Chinese speaking migrant mothers. Additional engagement with mothers specific to the program delivery could have elicited enhanced adaptations. A recent cultural adaptation of an obesity prevention program with fathers and older children (aged 5–12 years) provides an example of multiple ways to engage target program recipients in program adaptations [85].

The focus group qualitative data were intended to offer a range of perspectives from local Arabic and Chinese speaking migrant mothers. While there are benefits of group dynamics, individual interviews may have offered greater insights, acknowledging the diverse experiences of migration and culture. Individual interviews were not possible due to funding and time constraints. The careful steps taken when analysing the data (professional transcription and translation, multiple checking by bi-cultural staff, collective input from the diverse author team) aimed to maintain integrity and credibility of the analysis.

## Conclusion

The cultural adaptation of Healthy Beginnings followed an established process and resulted in a culturally adapted program informed by current evidence and local data. This work informs the subsequent stages of testing, refining and trialling the culturally adapted Healthy Beginnings program. Thorough reporting of the nature and context of this cultural adaptation offers insights for future cultural adaptations of evidence-based maternal and child health promotion programs.

## Supplementary Information

**Additional file 1.** Project team. A summary of the Healthy Beginnings cultural adaptation project team and contributors.

**Additional file 2.** FRAME adaptations and modifications. FRAME check list (Table 1) and brief report (Table 2) for the cultural adaption of Healthy Beginnings.

**Additional file 3.** Project timeline. Healthy Beginnings cultural adaptation project timeline.

**Additional file 4.** Focus group and interview brief surveys and guides. Brief demographic surveys and interview guides used for the individual interviews with health professionals and focus groups with mothers in Stage 2.

**Additional file 5.** Group characteristics. Group characteristics of mainstream Healthy Beginnings participants and proposed Arabic and Chinese speaking participants.

**Additional file 6.** Further illustrative participant quotes. Interview and focus group participant quotes to further illustrate the qualitative analysis findings.

**Additional file 7.** Sample content from culturally adapted booklets. Sample content from English, Arabic and Simplified Chinese Healthy Beginnings information booklets to illustrate cultural adaptations undertaken.

## Data Availability

The de-identified data that support the findings of this study may be available upon reasonable request to the corresponding author SM and with ethics approval.

## Abbreviations

CHAT: Communicating Healthy Beginnings Advice by Telephone
BMI: Body mass index
FRAME: Framework for Reporting Adaptations and Modifications-Enhanced
RCT: Randomised controlled trial
SMS: Short message service
