# Supplementary material for: The process of culturally adapting the Healthy Beginnings early obesity prevention program for Arabic and Chinese mothers in Australia
Source: BMC Public Health. 2021 Feb 4;21:284. doi: 10.1186/s12889-021-10270-5 (PMC7863271; doi:10.1186/s12889-021-10270-5)
Supplement: Supplementary file 1 — Additional file 1. Project team. A summary of the Healthy Beginnings cultural adaptation project team and contributors. [file 12889_2021_10270_MOESM1_ESM.docx]

**Additional file 1.**

**Healthy Beginnings cultural adaptation project team and contributors**

**PROJECT TEAM**

| Name | **A/Prof Li Ming Wen** |
| --- | --- |
| Project Role | **Principal investigator**  Lead project management and oversight  Conception, design and development of the project  Supervision of data analysis and reporting |
| Occupation | Director Population Health Research & Evaluation Hub - Sydney Local Health District  Adjunct A/Professor of Public Health - The University of Sydney  Adjunct Professor - School of Public Health, Fudan University, China |
| Credentials | MD, MMed, PhD |
| Background & experience | Li Ming Wen has extensive research experience in the fields of medicine, social science and public health in China and Australia. His research interests include clinical trials and practice and with expertise in evaluation of public health initiatives. |

| Name | **Prof Chris Rissel** |
| --- | --- |
| Project Role | **Senior investigator**  Contribution to project management and oversight  Conception, design and development of the project  Supervision of data analysis and reporting |
| Occupation | Director of the NSW Office of Preventive Health  Adjunct Professor of Public Health - The University of Sydney |
| Credentials | PhD |
| Background & experience | Chris’s work at the NSW Office of Preventive Health focuses on childhood and adult obesity prevention. His current research interests focus on obesity prevention and active transport, cycling advocacy, tobacco control and sexual health. |

| Name | **Prof Louise Baur** |
| --- | --- |
| Project Role | **Senior investigator**  Conception, design and development of the project  Supervision of data analysis and reporting |
| Occupation | Associate Dean of The Children’s Hospital at Westmead Clinical School  Professor of Child & Adolescent Health - The University of Sydney |
| Credentials | PhD, MD |
| Background & experience | Louise is a consultant paediatrician at the Sydney Children’s Hospitals Network where she is an active member – and former Head - of Weight Management Services, a multidisciplinary clinical service for children and adolescents with obesity. |

| Name | **Dr Sarah Taki** |
| --- | --- |
| Project Role | **Investigator**  Conception, design and development of the project  Supervision of data collection, analysis and reporting |
| Occupation | Senior Research Officer & Postdoctoral Fellow, Health Promotion Unit - Sydney Local Health District |
| Credentials | Bachelor of Nutrition and Dietetics, PhD |
| Background & experience | Sarah has expertise in qualitative research and m-health interventions. Her current work investigates conducting research for the implementation of the Healthy Beginnings program in the real-world setting. |

| Name | **Ms Marianne Kearney** |
| --- | --- |
| Project Role | **Project Coordinator**  Overall coordination of project implementation, data collection |
| Occupation | Project Coordinator, Health Promotion Unit - Sydney Local Health District |
| Credentials | Bachelor of Architecture, Graduate Diploma of Communications |
| Background & experience | Experience in research translation in Asia-Pacific region and developing and evaluating health promotion materials for nutrition and maternal health programs. |

| Name | **Ms Sarah Marshall** |
| --- | --- |
| Project Role | **Project Officer and PhD researcher**  Implementation of the research project, data collection, data analysis, reporting |
| Occupation | PhD Candidate, Sydney School of Public Health - The University of Sydney  Project Officer, Health Promotion Unit - Sydney Local Health District |
| Credentials | Bachelor of Nutrition and Dietetics (Hons), MSc (Global Health) |
| Background & experience | An accredited practicing dietitian with experience in community and public health and cross-cultural research. English-speaking and born in Australia. |

| Name | **Nancy Tam** |
| --- | --- |
| Project Role | **Health Promotion Officer/Project Officer**  Implementation of the research project, data collection |
| Occupation | Project Officer, Health Promotion Unit - Sydney Local Health District |
| Credentials | Bachelor of Nursing, Master of Health Personnel Education |
| Background & experience | Extensive experience working with culturally and linguistically diverse communities in Australia. Extensive experience in conducting research projects. Speaks fluent English, Cantonese and Mandarin |

| Name | **Sally Zhang** |
| --- | --- |
| Project Role | **Chinese bi-cultural research nurse**  Implementation of the research project |
| Occupation | Child and family health nurse and clinical nurse specialist - Sydney Local Health District |
| Credentials | Bachelor of Nursing, Grad Cert. Child and Family Health Nursing |
| Background & experience | Seven years of work experience as a clinical nurse specialist. Of Chinese background and speaks fluent English and Mandarin. |

| Name | **Julia Shadid** |
| --- | --- |
| Project Role | **Arabic bi-cultural research nurse**  Implementation of the research project |
| Occupation | Child and family health nurse - Sydney Local Health District |
| Credentials | Bachelor of Nursing, Grad Cert. Child and Family Health Nursing |
| Background & experience | Two years of work experience as a child and family health nurse. Of Lebanese background and speaks fluent English and Arabic. |

**FOCUS GROUP FACILITATORS**

The Chinese-Mandarin language focus groups were facilitated by Nancy Tam (project team) who was born in Hong Kong and speaks Mandarin, Cantonese and English.

The Arabic language focus groups were facilitated by Marial Sabry, Amal Tawfik and Seham Gerges, community health staff who were born in Egypt and speak Arabic and English.

All facilitators were experienced in facilitating groups and were migrants to Australia themselves.

**CONTRIBUTORS TO THE ADAPTATION PROCESS**

**Diversity Hub - Sydney Local Health District**

Denise Voros - Acting Coordinator - Cultural Support Program, Central and Eastern Sydney

Barbara Luisi - Director of Diversity Program and Strategy Hub

Nadia Matti – Cultural support worker

Layla Naji - Cultural support worker

Nevine Gayed - Cultural support worker

Elsi Samano- Cultural support worker

Faten Solaqa - Cultural support worker

**Sydney Healthcare and Interpreter Service**

Vesna Dragoje – Director

Agnes Lauder – Manager

Samia Labib – Arabic translator

**Health Promotion Unit - South Western Sydney Local Health District**

Marial Sabry – Arabic bi-cultural multicultural support worker

**South Eastern Sydney Local Health District**

Seham Gerges – Arabic bi-cultural multicultural support worker

**External Partner Organisations**

Chinese Australian Services Society

Lebanese Muslim Association

Advance Diversity Services

Diversity Kids

NSW Refugee Health Service

Juntos Marketing

**Mainstream** **Healthy Beginnings Project Team**

Li Ming Wen- Chief Investigator

Chris Rissel - Director NSW Office of Preventive Health and Professor, University of Sydney

Louise Baur- Professor of Child & Adolescent Health Associate Dean & Head WCH

Myna Hua- Manager Health Promotion Service, South Eastern Sydney Local Health District

Alison Hayes - Associate Professor in Health Economics

Catherine Llewellyn - Child and Family Health Clinical Nurse Educator

Paola Gordon - Child and Family Health Nurse Manager

Jenni Jones - Child and Family Health Clinical Nurse Consultant

Huilan Xu - Research & Evaluation Officer

Sarah Taki - Senior Research Officer

Mahalakshmi Ekambareshwar - PhD Candidate

Linda Elbayeh - Research Assistant

Christine Phillis - Research Nurse

Annmaree Lavery - Research Nurse

Wendy Smith - Research Nurse

Trisha Cant - Research Nurse

Angela Balafas - Communication Manager

Karen Bedford - Program Manager

Wenjie Liao - Chinese bi-cultural research assistant

**Mainstream Healthy Beginnings Advisory Committee**

Dr Teresa Anderson - Chief Executive, Sydney Local Health District

Lou-Anne Blunden - Director, Clinical Services Integration, Sydney Local Health District

Deborah Willcox - Director of Operations & General Manager, Royal Prince Alfred Hospital, Sydney Local Health District

Miranda Shaw - General Manager, Community Health, Sydney Local Health District

Renee Moreton, General Manager, Population Health, Sydney Local Health District

Julie Dixon - Director, Population Health, South East Sydney Local Health District

Julie Mooney - Executive Director, Nursing & Midwifery, Southern NSW Local Health District

Lorraine Dubois - Manager, Population Health. NSW Local Health District

Elisabeth Murphy - Senior Clinical Advisor Child Health, NSW Ministry of Health

Dr Jon Hyett - Prof Obstetrics and Gynaecology, Royal Prince Alfred Hospital

Kyra Sim - Manager Childhood Obesity, Sydney Local Health District

Philayrath Phongsavan - Prevention Research Collaboration, University of Sydney

Mandy Williams - Director of Health Promotion, South West Sydney Local Health District

Karen Wardle - Program Manager, South West Sydney Local Health District

Libby Powell – Manager, Health Promotion Service, Directorate Planning, Population Health and Equity, South Eastern Sydney Local Health District
