# Supplementary material for: The process of culturally adapting the Healthy Beginnings early obesity prevention program for Arabic and Chinese mothers in Australia
Source: BMC Public Health. 2021 Feb 4;21:284. doi: 10.1186/s12889-021-10270-5 (PMC7863271; doi:10.1186/s12889-021-10270-5)
Supplement: Supplementary file 2 — Additional file 2. FRAME adaptations and modifications. FRAME check list (Table 1) and brief report (Table 2) for the cultural adaption of Healthy Beginnings. [file 12889_2021_10270_MOESM2_ESM.docx]

## **Additional file 2.**

### Table 1: FRAME Check List for cultural adaptations of Healthy Beginnings

| **Process** |  |
| --- | --- |
| **When did the modification occur?**   - Pre-implementation/planning/pilot - Implementation - Scale up - Maintenance/Sustainment   **Were adaptations planned?**   - Planned/Proactive adaptation - Planned/Reactive adaptation - Unplanned/Reactive modification   **WHO participated in the decision to modify?**   - Political leaders - Funder - Organizational unit/team - Tx developer/purveyor - Administrator(s) - Treatment/intervention team - Provider - Program staff - Community members - Coalition - Recipient - Other________________   **Optional: Indicate who made the ultimate decision_**Organisational unit/team – Healthy Promotion Unit, Sydney Local Health District.  ***WHAT* is modified?**   - Content - Context - Training and Evaluation - Implementation and scale-up activities   **Contextual modifications are made to which of the following?**   - Format - Setting - Personnel - Population | **At what *LEVEL OF DELIVERY* (for whom/what is the modification made?)**   - Individual - Target Intervention Group - Cohort - Individual practitioner - Clinic/unit level - Organization - Network System/Community   **What is the *NATURE* of the content modification?**   - Tailoring/tweaking/refining - Changes in packaging or materials - Adding/Removing/skipping elements - Shortening/condensing/Lengthening/ extending (pacing/timing) - Substituting/Reordering/Spreading of intervention modules or segments - Integrating parts of the intervention into another framework - Integrating another treatment into EBD - Repeating elements or modules - Loosening structure - Departing from the intervention (“drift”) followed by a return to protocol within the encounter - Drift from protocol without returning   **Relationship to fidelity/core functions?**   - Fidelity Consistent/Core elements or functions preserved - Fidelity Inconsistent/Core elements or functions changed - Unknown |

| **Rationale** |  |
| --- | --- |
| **What was the goal?**   - Increase reach or engagement - Increase retention - Improve feasibility - Increase satisfaction | - Improve fit with recipients   --To address cultural factors   - Improve effectiveness/outcomes - Reduce cost |
| **Reasons**  **Socio-political**   - Existing Laws - Existing Mandates - Existing Policies - Existing Regulations - Political Climate - Funding Policies - Historical Content - Societal/Cultural Norms - Funding or Resource Allocation/ Availability   **Organization/Setting**   - Available resources (funds, staffing, technology, space) - Competing demands or mandates - Time constraints - Service structure - Location/accessibility - Regulatory/compliance - Billing constraints - Social context (culture, climate, leadership support) - Mission - Cultural or religious | **Provider**   - Race - Ethnicity - Sexual/gender identity   🗹 First/spoken languages   - Previous Training and Skills - Preference - Clinical Judgement - Cultural norms, competency - Perception of intervention   **Recipient**   - Race; Ethnicity - Gender identity - Sexual Orientation - Access to resources - Cognitive capacity - Physical capacity - Literacy and education level - First/spoken languages - Legal status - Cultural or religious norms - Comorbidity/Multimorbidity - Immigration Status - Crisis or emergent circumstances - Motivation and readiness - Other______________________ |

**References/sources:**

Stirman, S. W., Baumann, A. A. and Miller, C. J. (2019) ‘The FRAME: An expanded framework for reporting adaptations and modifications to evidence-based interventions’, *Implementation Science*. [doi: 10.1186/s13012-019-0898-y](https://implementationscience.biomedcentral.com/articles/10.1186/s13012-019-0898-y)

Checklist downloaded from: Stanford Medicine (2020), accessed 31 January 2020. <http://med.stanford.edu/fastlab/research/adaptation.html>

### Table 2: Brief report of Health Beginnings cultural adaptations based on FRAME elements

| **FRAME Elements** | **Brief report from culturally adapted Healthy Beginnings** |
| --- | --- |
| **Process** |  |
| **When did the modification occur?** | Cultural adaptations were made prior to implementation, based on formative research, community and stakeholder input; during stages 1 & 2 of the cultural adaption process undertaken. |
| **Were adaptations planned?** | Adaptations were primarily planned and proactive, based on the first two stages of the project (information gathering and preliminary adaptations). Planned/reactive iterative modifications during the implementation phase were through individual nurse calls whereby the script format was adjusted to suit individual participant needs. |
| **Who participated in the decision to modify?** | The project was led by the Health Promotion Unit, Sydney Local Health District research team, who made the ultimate decision to culturally adapt Healthy Beginnings. Many partners contributed to the decision to modify and were involved in making the cultural adaptations. This included many partners within the Sydney Local Health District, South Eastern Sydney Local Health District, community members, cultural community organisations and bi-cultural support workers. |
| **Adaptations** |  |
| **What was modified?** | Program content and context were culturally adapted. Refer to the tables within the manuscript comparing the mainstream and culturally adapted program content and delivery features. Training was offered to staff in a similar manner to the mainstream, but evaluation was modified. Evaluation was conducted with all intervention participants by bi-cultural research staff in-language. The mainstream survey was modified, professionally forward translated, pilot tested twice in language, refined and finalised. |
| **At what level of delivery were modifications made?** | Target Intervention Group - tailored to participant within nurse calls; Cohort - new mothers who have migrated from Arabic or Chinese speaking countries (can read and speak Arabic or Mandarin). |
| **What was the type or nature of context or content-level modifications?** | Refer to the tables within the manuscript comparing the mainstream and culturally adapted program content and delivery features. Contextual changes included working with bi-cultural personnel. The population changed from a general English-speaking population to Arabic and Chinese speaking population. The format/mode and setting were unchanged. Content changes included: Tailoring/tweaking/refining – maintaining the program principles while making the intervention more appropriate, applicable or acceptable - cultural adaptations, changes to language, different versions of program resources. At times nurse calls were extended, and call script was not followed directly, but was used as a guide. |
| **What was the relationship to fidelity?** | Efforts were made to critically consider and preserve core elements of the program (i.e. mode of delivery and key infant feeding and physical activity education messages, connecting mothers to community and health supports). |
| **Rationale** |  |
| **a) what was the goal? b) what were the reasons?** | a) Cultural modifications to improve fit among different cultures  b) High migrant population in Sydney, high proportion of Arabic and Chinese languages spoken. Need identified during mainstream program implementation. Arabic and Chinese speaking mothers with different modifiable risk factors for childhood obesity. Lower access to resources and support. Funding available through existing grant; state and local policies to reduce inequities and engage minority groups. Existing program implemented, resources and structures in place to support the adaptation project. |

**Reference:** Stirman, S. W., Baumann, A. A. and Miller, C. J. (2019) ‘The FRAME: An expanded framework for reporting adaptations and modifications to evidence-based interventions’, *Implementation Science*. [doi: 10.1186/s13012-019-0898-y](https://implementationscience.biomedcentral.com/articles/10.1186/s13012-019-0898-y)
