# Supplementary figures and images for: The process of culturally adapting the Healthy Beginnings early obesity prevention program for Arabic and Chinese mothers in Australia
Source: BMC Public Health. 2021 Feb 4;21:284. doi: 10.1186/s12889-021-10270-5 (PMC7863271; doi:10.1186/s12889-021-10270-5)

## **Additional File 3.**

### Healthy Beginnings cultural adaptation project timeline


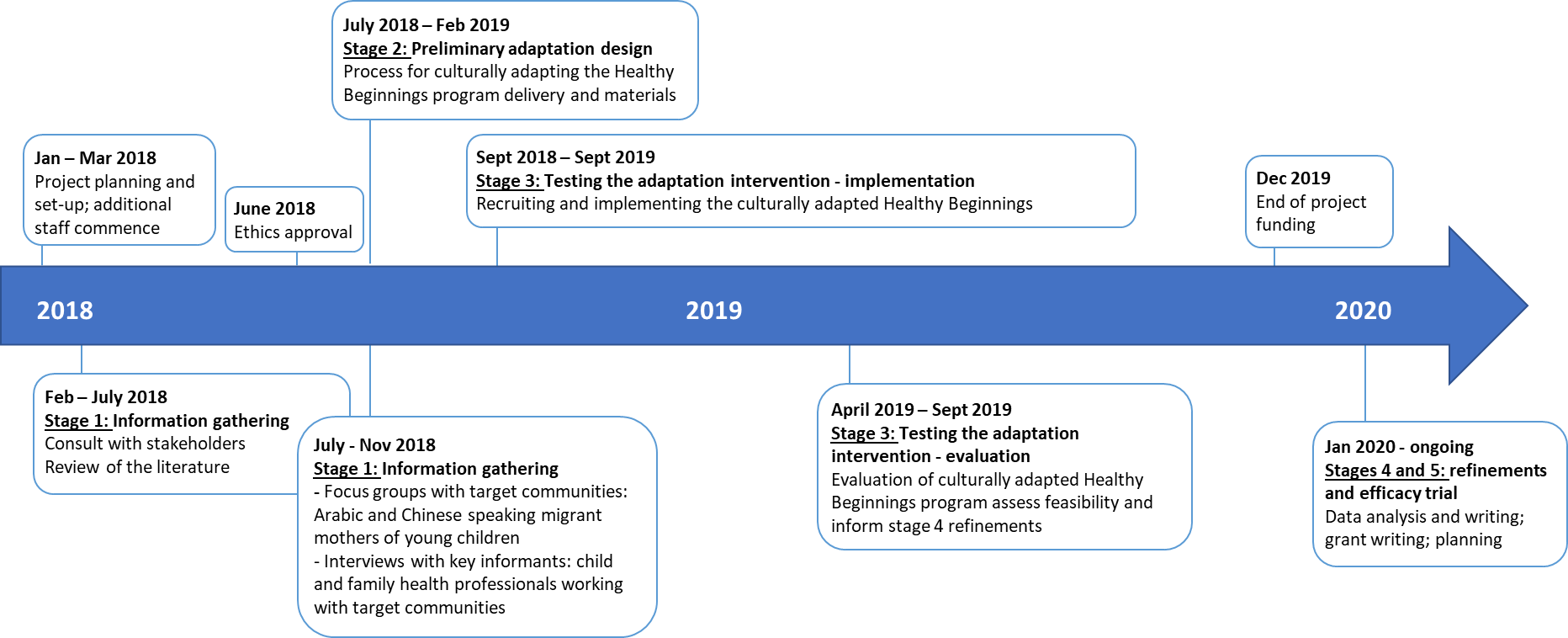

Supplement: Supplementary file 3 — Additional file 3. Project timeline. Healthy Beginnings cultural adaptation project timeline. [file 12889_2021_10270_MOESM3_ESM.docx]
