# Supplementary material for: The process of culturally adapting the Healthy Beginnings early obesity prevention program for Arabic and Chinese mothers in Australia
Source: BMC Public Health. 2021 Feb 4;21:284. doi: 10.1186/s12889-021-10270-5 (PMC7863271; doi:10.1186/s12889-021-10270-5)
Supplement: Supplementary file 4 — Additional file 4. Focus group and interview brief surveys and guides. Brief demographic surveys and interview guides used for the individual interviews with health professionals and focus groups with mothers in Stage 2. [file 12889_2021_10270_MOESM4_ESM.docx]

**Additional file 4.**

**Brief demographic surveys and interview and focus-group semi-structured discussion guides**

**Interviews with Health Professionals – Brief Demographic Survey**

These questions are about your work, work history, and language. You will not be identified (we do not ask your name here) and your answers will be kept confidential.

1. What is your current role? *(Please specify)*
2. In which Local Health District(s) do you work?

Sydney Local Health District

South Eastern Sydney Local Health District

South Western Sydney Local Health District

Other, please specify

1. How long have you worked in this role? *(specify approximate months or years)*

months OR years

| 1. Do you have experience in Australia working with the following client groups? |  |  |
| --- | --- | --- |
| *(tick all that apply. If ticked, please answer questions in next two columns)* | Are you currently working with this group? | Months/years of experience working with this group? |
| Mandarin-speaking mothers of young children (0-5 years) | Yes  No | months  years |
| Mandarin-speaking community | Yes  No | months  years |
| Arabic-speaking mothers of young children (0-5 years) | Yes  No | months  years |
| Arabic-speaking community | Yes  No | months  years |

1. What language do you normally speak at home? (*tick one box, and specify language*)

Only

*e.g. English*

Mostly

English and other

**Box 1: Interview discussion guide (health professionals)**

| *Introduction*   1. Tell me about your role and your experiences with [Chinese/Arabic] mothers and families? 2. Can you describe the background of the families that you see from the [Chinese/Arabic] community? – How long in Australia? Level of education and literacy? Areas of residency?   *Health information and services*   1. What do you think helps/prompts [Chinese/Arabic] mothers to seek health information/health services? 2. What might stop mothers from accessing health care or information? 3. In your experience, what are the best ways to engage with [Chinese/Arabic] mothers and families?    - *Best ways to get your messages across regarding mum and child’s health (diet, exercise and weight)? What would make health messages more acceptable?*   *During and after pregnancy, mother’s diet and exercise*   1. Are there any common beliefs or behaviours related to [diet/exercise] that are practised in [Chinese/Arabic] women before, during or after pregnancy? 2. What roles do partners/fathers and family have during pregnancy and after baby is born?   *Infant feeding*   1. Can you tell me about any specific attitudes/views about breastfeeding among women from [Arabic/Chinese] cultures? About exclusive breastfeeding until 6 months?  - *What influences breastfeeding decisions? Where/from who do [Chinese/Arabic] mothers commonly get advice and support about breastfeeding?*  1. Can you tell me about any specific attitudes/views about formula feeding among women from [Chinese/Arabic] cultures? is it commonly practised?  - *What influences formula feeding decisions? (To start? To use regularly?) Where/from who so [Chinese/Arabic] mothers commonly get advice about formula feeding?*  1. Can you tell me about any specific attitudes/views about the introduction of solid foods to infants of mothers from [Chinese/Arabic] cultures?  - *Around what age are solid foods first introduced? What types of foods are commonly first introduced? Do you know of any specific reason for choosing these first foods? How are they offered (by spoon; as finger foods)? When and what types of drinks are first introduced? and how?*   *Infant play/physical activity*   1. From your experience, what are [Chinese/Arabic] mothers’ attitudes to active play among children (0-2 years)? Is this the same/different as partners/fathers?  - *Is tummy-time practiced? Are you aware of any specific attitudes/views about tummy time?*  1. From your experience, what are [Chinese/Arabic] mothers’ attitudes/behaviours around young children’s (0-2 years) screen-time (tv, DVD, computer, tablet, other devices)?    - Is this the same/different as partners/fathers or other family?   *Infant weight*   1. Have you noticed or experienced any attitudes among the [Chinese/Arabic] community about an overweight baby or the size of baby?  - *Is being overweight perceived as a problem or a concern? Are there any characteristics that mothers or community want/expect for a healthy baby?*   Are there some things we have not covered that you would like to add?  Thank you again for agreeing to take part in this interview. We appreciate your expertise and input. |
| --- |

**Focus Group with Mothers – Brief Demographic Survey**

These questions are about your background, age, language and education. You will not be identified (we do not ask your name here) and your answers will be kept confidential.

1. In which country were you born? *(tick one box)*

- Australia
- Other, please specify _____________________

1. How many years have you been living in Australia? *(write number of years)*

______years, OR € Always lived in Australia

1. What is your age? *(tick one box)*

| - 16-19 - 20-24 - 25-29 - 30-34 | - 35-39 - 40-44 - 45-49 - 50+ |
| --- | --- |

1. What is the highest qualification you have completed? *(tick one box)*

- No schooling
- Not completed primary school
- Completed primary school
- Completed years 7 to 9
- Completed School Certificate or Intermediate Certificate or Year 10 or 4th Form
- Completed High School Certificate or Leaving Certificate or Year 12 or 6th Form
- TAFE certificate or diploma
- University or some other tertiary institute degree or higher
- Other, please specify _______________________________

1. What language do you normally speak at home? (*tick one box only*)

- Only [Mandarin/Arabic]
- Mostly [Mandarin/Arabic]
- English and other [Mandarin/Arabic]
- Mostly English
- Only English

1. a) What ethnic group do you identify with? (*tick one box only*)

- Only another ethnic group
- Mostly another ethnic group
- Australian and another ethnic group equally
- Mostly Australian
- Only Australian

b) If another ethnic group, please specify ____________________________

1. What is your religion? (*tick one box only*)

- No religion
- Christian
- Islam
- Judaism
- Buddhism
- Other, please specify ____________________________

Thank you for completing this survey and

agreeing to take part in the group discussion.

**Box 2. Focus group discussion guide (Arabic or Chinese speaking mothers with children aged 0-5 years)**

| *Conducted by bi-cultural workers in Arabic or Chinese-Mandarin language*  **Part 1: Experiences related to feeding and activity of baby** (approx. 60 mins)   \| Mothers’ diet and exercise \| 1. Thinking back when you were pregnant, were there any special foods you ate or didn’t eat around the time you were pregnant? Can you tell me about these? [*allow group responses*] \| \| --- \| --- \| \|  \| 1. Again, thinking back to when you were pregnant, were there any special or specific exercises/physical activities that you did or didn’t do around the time you were pregnant? Can you tell me about these? [*allow group responses*] \| \|  \| 1. What was the most useful information or advice you received while you were pregnant? Where from? [*allow group responses*] \| \| Infant feeding \| 1. Are there any cultural expectations on you as a mother? [*What does the community expect or value?]* \| \|  \| 1. Thinking back to when your child was very young (0- 6 months old), can you tell me about your early experiences feeding them? [*allow group responses*] \| \|  \| *Breastfeeding:*   1. Did any of you breastfeed any of your children? Why did you decide to do this? For how long? Why did you stop early (less than 6months)? Why did you feed for longer (>6 months)? 2. Are there any views about breastfeeding within your community - describe? 3. Where/from who did you get specific advice about breastfeeding?   *Formula feeding*   1. Did any of you formula-feed any of your children? Why did you decide to do this? For how long? Why? 2. Are there any views about formula feeding within your community - describe? 3. Where/from who did you get specific advice about formula feeding? \| \|  \| 1. Thinking back to when you started to introduce your baby to solid foods (such as mashed or cooked fruits or cereals), can you tell me about your experiences/what happened for you? [*allow group responses*] 2. How old was your baby? What made you decide to start feeding solids? 3. Where/from who did you get specific advice about feeding solids? 4. What foods were first introduced? Any specific reason for choosing these first foods? How did you offer these foods (by spoon; as finger foods)? 5. When did you start to offer drinks? What drinks were first introduced? how? \| \| Infant activity & screen time \| 1. Thinking back to when your child was very young (0-6 months old), can you tell me about any specific advice you received about your baby’s physical activity/play time? Where from? \| \| 1. What do you think of these photos? [Arabic/Chinese babies doing tummy time] Is this something you would do/did do with your baby? Why/why not? 2. Within your family, community, and/or culture, are there any views about doing these kinds of activities with your baby - describe? \| \| 1. Thinking back to when your child was 0-6 months old, can you tell me about their interactions/use with screens - TV, DVD, computer, phones, tablets or other devices? \| \| Infant weight \| 1. What a healthy child is to you - can you describe?   e.g. characteristics, behaviours, physical appearance \|   **--- tea break** (10-15 mins) **---**  **Part 2: Feedback on draft Healthy Beginnings booklets** (approx. 20 mins) |
| --- | --- | --- | --- | --- | --- | --- | --- | --- | --- | --- | --- | --- | --- | --- | --- | --- | --- | --- | --- | --- |
