# Supplementary material for: The process of culturally adapting the Healthy Beginnings early obesity prevention program for Arabic and Chinese mothers in Australia
Source: BMC Public Health. 2021 Feb 4;21:284. doi: 10.1186/s12889-021-10270-5 (PMC7863271; doi:10.1186/s12889-021-10270-5)
Supplement: Supplementary file 5 — Additional file 5. Group characteristics. Group characteristics of mainstream Healthy Beginnings participants and proposed Arabic and Chinese speaking participants. [file 12889_2021_10270_MOESM5_ESM.docx]

## **Additional file 5.**

### Group characteristics of mainstream Healthy Beginnings (CHAT) participants and proposed Arabic and Chinese speaking population

|  | **Mainstream Healthy Beginnings CHAT participants – English speaking mothers** | **Cultural participant group (1) - Arabic speaking migrant mothers** | **Cultural participant group (2) – Chinese speaking migrant mothers** | **Potential mismatch** |
| --- | --- | --- | --- | --- |
| **Characteristics** |  |  |  |  |
| **Primary language** | English | Arabic | Mandarin-Chinese | Language differences may impact understanding and access to intervention |
| **Ethnicity** | White, mainstream population | Ethnic minority | Ethnic minority | Differences in social norms, attitudes, beliefs, cultural factors |
| **Socioeconomic position (SEP)** | Lower SEP | - | - | Potentially no difference in SEP |
| **Setting** | Urban - Greater Sydney | Urban – Greater Sydney | Urban – Greater Sydney | n/a |
| **Country of birth** | Australia, or other English-speaking country | Outside of Australia | Outside of Australia | Differences in life experiences, social norms, attitudes, beliefs, cultural factors |
| **State of migration** | Generally established | Recent migration | Recent migration | Differences in migration status, does not account for migration as a health determinant |
| **Access to universal healthcare** | Access | Lower access. Impacted by factors such as language and understanding the system | Lower access. Impacted by factors such as language and understanding the system | Does not account for reduced health service access and differences in health literacy |
| **Childhood obesity risk** | Lower for Australian-born, English-speaking | Higher than mainstream population | Higher than mainstream population | Unknown effect on behavioural risk factors due to existing program eligibility criteria |
| **Staff** |  |  |  |  |
| **Type of staff** | Trained staff, primarily English-speaking | - | - | Unable to speak mothers’ primary language.  Potentially limited awareness of, or sensitivity to, cultural issues |
| **Community engagement** |  |  |  |  |
| **Community consultation** | Consultation with experts in program design and  delivery | - | - | No cultural community engagement, therefore potential of no community “buy-in” |
