# Supplementary material for: The process of culturally adapting the Healthy Beginnings early obesity prevention program for Arabic and Chinese mothers in Australia
Source: BMC Public Health. 2021 Feb 4;21:284. doi: 10.1186/s12889-021-10270-5 (PMC7863271; doi:10.1186/s12889-021-10270-5)
Supplement: Supplementary file 6 — Additional file 6. Further illustrative participant quotes. Interview and focus group participant quotes to further illustrate the qualitative analysis findings. [file 12889_2021_10270_MOESM6_ESM.docx]

## **Additional file 6.**

**Participant quotes to further illustrate qualitative analysis findings**

Related to obesity-related behaviours and prevention among Arabic and Chinese speaking migrant mothers

| **Domains and sub-themes** | **Participants^^^** | **Illustrative quotes^#^** |
| --- | --- | --- |
| **Domain 1: Beliefs and practices related to infant obesity-related behaviours (infant feeding, active play, sedentary behaviour and sleep)** | | |
| Confinement practices important for recovery after birth | Ar, Ch, HP | *“In the old times, they used to tell us to stay 40 days at home after birth, according to Lebanese traditions. I didn’t like that as I wanted to go out and walk around….I wish if I could go out more, but my mother thought it was healthier if you always stayed at home.”* - Arabic speaking mother, Focus Group 4  *“Because you must do the proper thing during the maternity month. I thought I should be all right; I was still very young. But everything was very traditional. She (sic) wanted to tell you what to eat, what you can eat, what not to eat. I did not want to listen to her. So it was quite unpleasant for me to go through the maternity month.”* Chinese speaking mother, Focus Group 2  *“It depends on the mother themselves whether they follow the, um, guideline here or that in their own country. You know, they, the feel will different here.”* - Multicultural worker, 6 years, Mandarin and English  *“And you understand sometimes with home visits if they need follow up, rather than making them come to clinic because often they have that period of confinement... You know, I will just offer them a follow-up home visit …I think they feel a lot more welcomed and accepted that way.”* - Child and family health nurse, 14 years, English |
| Breastfeeding as part of the social norm and expected | Ar, HP | *"Frankly, non-one advised me to bottle-feed, all people encouraged me to breastfeed." -* Arabic speaking mother, Focus Group 4  *“I’m upset because I couldn’t breastfeed and that is not good at all "* - Arabic speaking mother, Focus Group 6  "*They [Arabic mums] really aim to breastfeed for two years because I think it's in the Qur’an…There seems to be a lot more support amongst each other. For the breastfeeding, because, they want everybody to breastfeed for that, so I feel that their community is perhaps more supportive of it."* - Child and family health nurse, 23 years, English  "*The Arabic population just sort of get on with it [breastfeeding]."* - Child and family health nurse, 3 years, English |
| Not enough breastmilk and formula use for reassurance | Ch, HP | "*At that time, my mother was helping me with the baby, so when the baby was hungry, you would not necessarily have enough breast milk."* -Chinese speaking mother, Focus Group 1  "*Up to week 10, I was still breastfeeding. Then I was not producing enough breast milk, so I decided to breastfeed in the evening, and add more [feeds]."* - Chinese speaking mother, Focus Group 2  *“[Among Chinese mums] there is a very low level of confidence that her body can produce enough milk…The predominant attitude is that they can't produce enough milk."* - Child and family health nurse, 29 years, English  *“So Chinese women would be more likely to want to supplement, and would be open to thinking that powdered milk is better for the baby as well...There's always questions about - is my milk enough, do I have enough, is it got the right properties in it?”* - Multicultural worker, 3 years, English and Mandarin |
| Feeding as helpful for weight gain, sleep and settling | Ar, Ch, HP | *“My mother says: even if the child is full, she still insists to finish his food.”* - Arabic speaking mother, Focus Group 4.  *“The baby was still willing to suck, but he just could not get anything out. Each time, he would still be hungry. [Interviewer: How did you know that she was not full?]. I think, because every night I have 45 minutes feeding baby, and she still cries all the time.”* – Chinese speaking mother, Focus Group 4.  *“Some will give, a little, the odd bottle, thinking they might sleep better.”* - Child and family health nurse, 28 years, English  *"If they whinge, you give them food or milk because they will stop complaining, even if they might be crying over something else."* - Child and family health nurse, 23 years, English  *“I think their mothers feel more confident that they're providing the right food and all that sort of thing and also I think that they think the more food they eat and the earlier they eat it […] the better they're going to sleep ... the mothers, as well.”* - Child and family health nurse, 23 years, English |
| Confusion with timing of complementary feeding | Ar, Ch, HP | *“My mother said 4 months, and you should give solid foods as the milk alone doesn’t provide full [nutrition]. I started to give all my children boiled eggs with honey at the age of six months."* - Arabic speaking mother, Focus Group 4  *“You can then puree this thing for him to have a taste.* *If the child shows no interest, then you can maybe wait. If he shows interest at a fairly early stage, you can try it early with him.”* - Chinese speaking mother, Focus Group 1  *“My mother said I should start giving baby all sorts of food when he was 4 months old. I said no, he was too little. Just to give it a try, rice, soup, steamed rice, noodles…But I refused to give him.”* - Chinese speaking mother, Focus Group 2  *“I think most of the grandparents, they think natural food will be better [than adding formula]. Especially for like eggs and meat. Yeah, it will be good for the baby, so they will add extra food at very early stage.” -* Multicultural worker, 3 years, English and Mandarin  *“Solid introduction, that again very much depends on the advice from their family members. And again, there have been some of the taking advice more than us. From them rather than us. We've had some doctors again put them on to solids at three months. With that so you know, there's a lot of conflicting advice out there from paediatricians, from GPs. And then from the relatives, and we just try to keep this line of between five to six months, closer to six months.” -* Child and family health nurse, 14 years, English |
| Tummy time as a new concept | Ar, Ch, HP | *“We put the baby on his tummy on our laps but not on the floor. Putting him on our laps relieves the colic with comfort for his tummy.” -* Arabic speaking mother, Focus Group 5  *“We don’t put the baby on the floor [in Syria]. …This is the first time I know about it” -* Arabic speaking mother, Focus Group 5  *“They don’t seem to do this in China. Because my friends gave births in China, they would probably not be able to crawl at three months or 4 months.”* - Chinese speaking mother, Focus Group 1  *“I personally do not have any idea when to start this tummy time.”* - Chinese speaking mother, Focus Group 1  *“For the very little baby exercise, it's not like, a lot of the focus or priority for our mothers to do that. But it all depends on whether the mum has received those kinds of information prior to their delivery.”* - Multicultural worker, 6 years, Mandarin and English  *“And then there's nothing called tummy time in our days. In our days mean uh in the Chinese culture. It's only in the Western culture.” -* Child and family health nurse, 16 years, Cantonese and English  *“I think they are more likely to be holding baby and cuddling them” -* Child and family health nurse, 3 years, English  *“[Tummy time] that's a new concept also for them. Sometimes the mothers are not confident to do it, sometimes they don't know how to do it but we do role model it a lot.” -* Nejla, Child and family health nurse, 6 years, Arabic and English |
| The floor is not seen a place for new babies to play | Ar, Ch, HP | *“We don’t put the baby on the floor [in Syria]. …This is the first time I know about it”* - Arabic speaking mother, Focus Group 5  “*Because I could not approve it in my mind, so I did not do it….In the last month, we put a towel underneath*” - Chinese mother, Focus group 2  *“I'll demonstrate tummy time on the change table and say it is really important that you stay with the baby, but you can do tummy time up here. Because part of the Chinese culture is not to put the baby on the floor. So, if they can start tummy time on a change table and get the baby a bit stronger, then they might be able to progress to the floor.”* - Child and family health nurse, 29 years, English  *“The other one that's common is being reluctant to put their babies on the floor, to play.”* - Child and family health nurse, 30 years, English  *“For the tummy time, the Asian culture, they won't put the baby on the floor. So that's why there is a lot of cultural difference…The floor is cold and the floor is dirty so that's why not a lot of Chinese families will not be very eager or very happy to put the baby on the floor”* – Child and family health nurse, 16 years, Cantonese and English |
| Tummy time as a risk for new babies | Ch, HP | *“Someone in my family said that the baby was too young, and said that the spine was still not quite developed. Basically, he told me not to do this tummy time, so I seemed to wait until he was about 1 month old before I started to help him do this*.” - Chinese speaking mother, Focus Group 1  *“We put him down, he was able to raise his head. It was the first week…Then at the time we were having a video chat with my father in law, he said the baby was about to break his back (laughs).” -*  Chinese speaking mother, Focus group 1  *“Sometimes we'll actually talk about that. …I can acknowledge that you are going home, that the grandparents are not going to want you to do this… because the grandparents say - don't put the baby on their tummy until 6 months of age.”* - Child and family health nurse, 29 years, English |
| Interactions/play with babies as natural | Ar, Ch, HP | *“I let him recognize colours and movements with the music, I used to train him how to move his hands and legs, or I move something in front of him to watch, something like that. I also do movements in front of him like now look how is he watching my movements, and where and how did I put my hand.”* *–* Arabic speaking mother, Focus Group 5  *“My first baby was different from the others [subsequent babies], I pamper him, play with him… things like this, sing to him a lot”* *–* Arabic speaking mother, Focus Group 5  *“Starting from the maternity month. Whenever he was awake, I would read nursery rhymes to him. He did not make much sound, but it did not affect his sleep. But as soon as he is awake, I would talk about anything that I happened to see, anything I happened to do, just non-stop talking.”* *-* Chinese speaking mother, Focus Group 2  *“And in China, the teacher would help them massage, and tell the mums, pre-mums to massage their babies. It seems that over here there is no such specific requirement that asks you to do massage…Then he said through muscle development you can also build relationship with the baby.”* *-* Chinese speaking mother, Focus Group 2  *“Generally, the sort of things that they will do with the baby- they do hold the baby and talk to the baby… [I explain] for example how to hold the baby, um a bit away from your face so the baby can see you, um you know smiling, looking. Giving the baby a chance to say things back to you, they're often not getting this parenting advice in regards to communication with the baby”* - Child and family health nurse, 6 years, Arabic and English |
| Dilemmas of screen use | Ar, Ch, HP | *“it is good that child listens and talks, but not for continuous hours because it will cause vision problems in future”* Arabic speaking mother, Focus Group 5  *“In the first six months was no TV, the daily play depended more on songs and words that I say to him [my baby]” –* Arabic speaking mother, Focus Group 5  *“With my first two children, I used to be a perfect mother; but after that don’t ask. With the first two children no TV, nothing. They sit down and eat. But with my daughter [third child], phone, iPad, anything.”* Arabic speaking mother, Focus Group 4  *“I feel many mums would give an iPad to a child when the child is very small, to distract the child so that she can do her own things. I feel I’d rather give some Legos or some books. Or some other things along these lines.” -* Chinese speaking mother, Focus Group 1  *“My child has been playing the iPad, but he learned a lot of things.” -* Chinese speaking mother, Focus Group 1  *“All the information that I have read says only use it after 3 years old, otherwise it would affect the brain or the eyes.”* *-* Chinese speaking mother, Focus Group 3  “*They [Arabic community] are big screen watchers. They do believe that screen time for their children does make them smarter*” - Child and family health nurse, 6 years, Arabic and English  *“They use a lot of screen time you know as I said in our culture everyone in apartments. […] They use the screen time yes that's true. Like the Tablets, you know. DVD they use a lot. Because they believe that this is also the way that children can learn and as I said we are not used to go out to the park, 'cause no park available.”* Child and family health nurse, 16 years, Cantonese and English |
| Screens to distract baby while feeding | HP | *“Time and time again because you've got the importance of having a chubby child so they're wanting to get that child to have lots of food. The child will eat more if they're distracted at the screen, so it's very common. and the other thing is…because the family doesn't want the mess from finger food and all that sort of thing they often resort to the screens so the child will settle down, and … they feed the baby while the baby's distracted.”* - Child and family health nurse, 29 years, English  “*Some of the Chinese and the Arabic women they put them in front of, when they are on solids, they'll put them in front of there because they can spoon more food in.”* - Child and family health nurse, 23 years, English  *“I have seen people using screens to distract their children. I don't think it's [culturally specific]… I think it's just the whole of society”* - Child and family health nurse, 14 years, English |
| **Domain 2: Perceptions related to child weight** | | |
| A bigger baby as a healthier baby | Ar, Ch, HP | *“When the baby puts on weight this is a sign of good health” -* Arabic speaking mother, Focus Group 6  *"In Syria, the baby’s weight indicates his health. They are just concerned about the health and weight, they don’t pay attention to how much weight he put, e.g. 200 grams per week or so… if the baby is slim, they think he doesn’t have enough feeding" -* Arabic speaking mother, Focus group 3  *“Average would be fine. Should not be fat.” ; “But many people would prefer chubby, to feel healthier.” -* Chinese speaking mother, Focus group 3  *“A little bit overweight is a sign that they're well fed or a very responsible mum." -* Multicultural worker, 3 years, English and Mandarin  *“**The weight is important. It’s healthy. If they are skinny, then people will ask is there anything wrong with the baby.”* - Child and family health nurse, 16 years, Cantonese and English |
| Infant overweight as not a concern | HP | “*For our community, generally…we don't consider it overweight. We've always, sometimes we praise, "Oh, you've raised this baby so well! They're so chubby. They're so cute." But we don't see that this is a constant worry or, it's too fat.”* - Multicultural worker, 6 years, Mandarin and English  *“They don't like you saying your child is overweight. There doesn't seem to be an understanding of that. They're much more in tune with your child is under weight."* - Child and family health nurse, 14 years, English  *"Often when they come in to do their [routine health] checks, we do sometimes have to reassure them that they are growing, there's a range of normal, and … that fatness doesn't equal health."* - Child and family health nurse, 30 years, English  *“They do want bigger babies and often, the ones that are overweight, they don't seem to be concerned about them because they just think they're thriving and that they're being fed and that’s really great"* - Early childhood dietitian, 14 years, English  *“I think that sort of "fat baby" thing is more an infant thing; as they get a bit older, I don't think they necessarily perceive it that way.”* - Child and family health nurse, 14 years, English |

*^ Ar = Arabic speaking mothers (focus groups); Ch = Chinese speaking mothers (focus groups); HP = child and family health professionals (interviews)*

*# Health professionals are described using their role, their years of relevant experience and their main language(s) spoken; Mothers are described using their focus group language and their focus group number (1-6).*
