# Supplementary material for: The process of culturally adapting the Healthy Beginnings early obesity prevention program for Arabic and Chinese mothers in Australia
Source: BMC Public Health. 2021 Feb 4;21:284. doi: 10.1186/s12889-021-10270-5 (PMC7863271; doi:10.1186/s12889-021-10270-5)

## **Additional file 7.**

## **Sample content from English, Arabic and Simplified Chinese Healthy Beginnings booklets**

The Healthy Beginnings booklets align with baby age and the program’s key messages – focussing on infant feeding and active play. Available from [www.healthybeginnings.net.au](http://www.healthybeginnings.net.au)

This **sample content is from Healthy Beginnings information booklet targeting infant age 4-6 months, pages 4 and 5**. Content is focussed on introducing solids foods. English mainstream booklet (a), and culturally adapted Simplified Chinese (b) and Arabic (c) versions are displayed below.

Key changes in this sample include:

- Images include individuals with appearance of target populations
- Reduced and simplified text, reading level lowered <grade 6 (prior to translation)
- Increased visuals
- Adapted wording and translated into Arabic and Simplified Chinese languages
- Content is focussed on culturally relevant information
  - Emphasis on hunger and fullness cues (relates to belief of earlier introduction of complementary foods as a positive sign of baby development, and also relates to perceptions of less-responsive feeding)
  - Emphasis of baby making a mess and self-feeding (relates to perceptions of needing to maintain a clean space while baby is eating, and not self-feeding)
  - Image of sippy cup to illustrate what this is and promote cup use
  - Images of culturally relevant foods and consistencies for baby between 6-12m.

1. Mainstream English language information booklet targeting infant age 4-6 months.


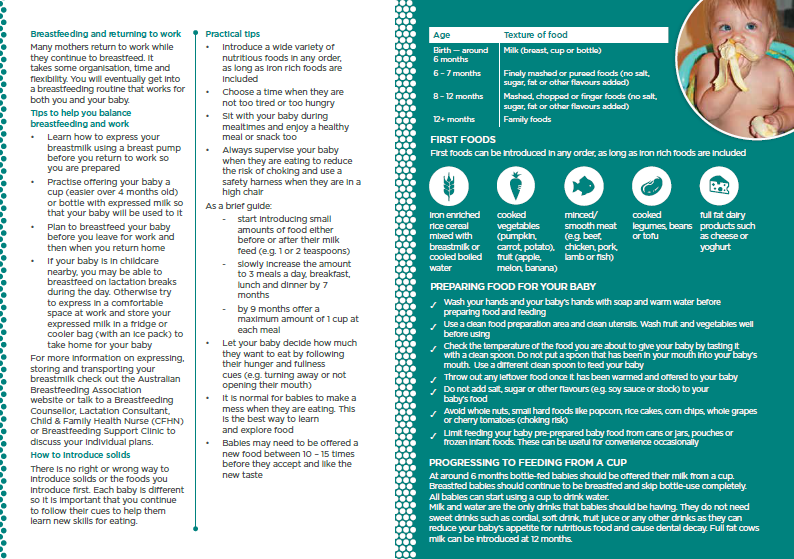


1. Culturally adapted Simplified Chinese language information booklet


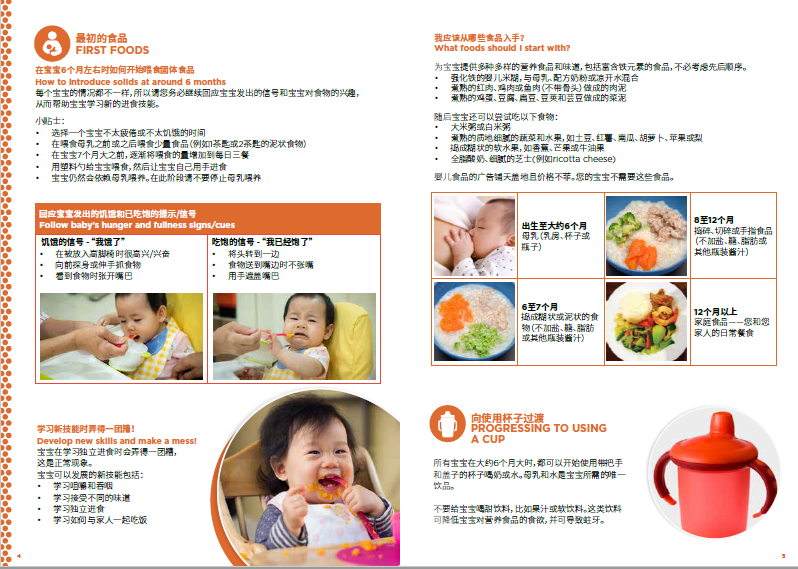


1. Culturally adapted Arabic language information booklet


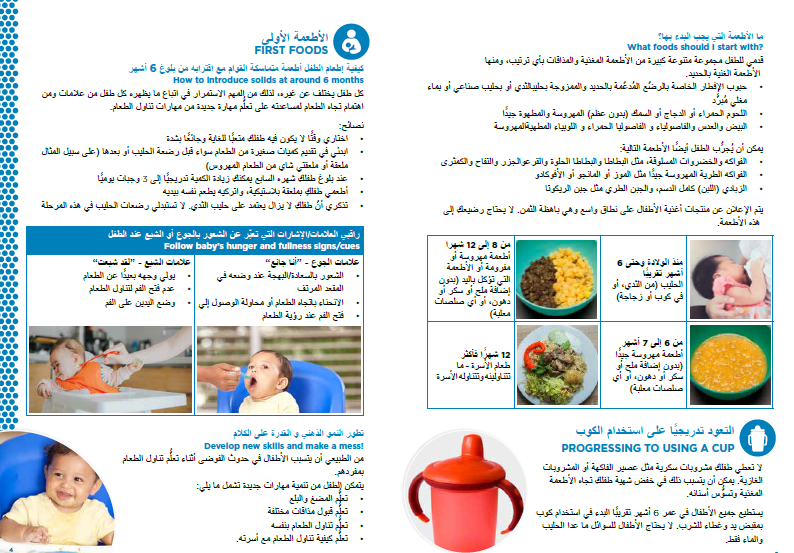

Supplement: Supplementary file 7 — Additional file 7. Sample content from culturally adapted booklets. Sample content from English, Arabic and Simplified Chinese Healthy Beginnings information booklets to illustrate cultural adaptations undertaken. [file 12889_2021_10270_MOESM7_ESM.docx]
